# Genome-wide characterization and expression profiling of *NAC* transcription factor genes under abiotic stresses in radish (*Raphanus sativus* L.)

**DOI:** 10.7717/peerj.4172

**Published:** 2017-12-15

**Authors:** Bernard Kinuthia Karanja, Liang Xu, Yan Wang, Everlyne M’mbone Muleke, Bashir Mohammed Jabir, Yang Xie, Xianwen Zhu, Wanwan Cheng, Liwang Liu

**Affiliations:** 1 National Key Laboratory of Crop Genetics and Germplasm Enhancement, Key Laboratory of Horticultural Crop Biology and Genetic Improvement (East China) of MOA, College of Horticulture, Nanjing Agricultural University, Nanjing, China; 2 Department of Plant Sciences, North Dakota State University, Fargo, ND, United States of America

**Keywords:** Abiotic stress, Expression profiling, Radish, Genome-wide analysis, NAC, Transcription factor

## Abstract

NAC (NAM, no apical meristem; ATAF, *Arabidopsis* transcription activation factor and CUC, cup-shaped cotyledon) proteins are among the largest transcription factor (TF) families playing fundamental biological processes, including cell expansion and differentiation, and hormone signaling in response to biotic and abiotic stresses. In this study, 172 RsNACs comprising 17 membrane-bound members were identified from the whole radish genome. In total, 98 *RsNAC* genes were non-uniformly distributed across the nine radish chromosomes. *In silico* analysis revealed that expression patterns of several *NAC* genes were tissue-specific such as a preferential expression in roots and leaves. In addition, 21 representative *NAC* genes were selected to investigate their responses to heavy metals (HMs), salt, heat, drought and abscisic acid (ABA) stresses using real-time polymerase chain reaction (RT-qPCR). As a result, differential expressions among these genes were identified where *RsNAC023* and *RsNAC080* genes responded positively to all stresses except ABA, while *RsNAC145* responded more actively to salt, heat and drought stresses compared with other genes. The results provides more valuable information and robust candidate genes for future functional analysis for improving abiotic stress tolerances in radish.

## Introduction

Radish (*Raphanus sativus* L.) is one of the most economically important root vegetables in Brassicaceae family. To date, several studies on the radish genome have been carried out in the past few years, which includes the recently released radish genome sequence of radish contributed significantly to further characterization of the genes in radish (Kitashiba et al., 2014; Mitsui et al., 2015). In the published sequence data, comprehensive analysis of a gene family can be performed to explore the phylogenetic evolution and expression patterns that will facilitate the understanding how NAC gene family controls traits at a genome-wide level. In radish, decreased yield and quality due to environmental stresses including HM, heat and high salt have been reported (Sun et al., 2016; Wang et al., 2015a; Xie et al., 2015). Therefore, plants tolerance to these stresses could contribute to higher yield and quality in radish production. Tolerance against stresses is regulated by several specific TFs, such as *NACs* (Vinocur & Altman, 2005). These TFs function together in regulating plant growth and development by activating or suppressing the transcriptional processes of their target genes (Udvardi et al., 2007). Recently, the NAC gene family has gained much attention due to its involvements in numerous functions such as the development of tolerance to environmental cues (Yang et al., 2011).

The NAC TF regarded as one of the largest TF families is composed of 117 in (*Arabidopsis*), 204 in Chinese cabbage (*Brassica oleracea*), 110 in potato (*Solanum tuberosum*), 104 in tomato (*Solanum lycopersicum*), 82 in melon (*Cucumis melo*), 74 in grape (*Vitis vinifera*), 151 in rice *(Oryza sativa*), 147 in foxtail millet (*Setaria italica*) and in 120 poplar (*Populus* Spp) *NAC* members (Hu et al., 2010; Liu et al., 2014; Nuruzzaman et al., 2010; Puranik et al., 2013; Singh et al., 2013; Wang et al., 2013a; Wei et al., 2016). In addition, several NAC proteins have been associated with plant defense against stress conditions. For example, high salinity (Zheng et al., 2009), drought (Jeong et al., 2010) and HMs (Wang et al., 2013b; Xu et al., 2013). Evidently, NAC family genes are critical regulator implicated in the survival of plants under various stress conditions. In *Arabidopsis,* expression levels of three *NAC* genes (*ANAC072, ANAC055* and *ANAC019*) were found to be elevated under ABA, salt and drought stress conditions. Furthermore, overexpression of these genes increased abiotic stress tolerance as compared to the wild-type (Hu et al., 2006; Jensen et al., 2010). Similarly, transgenic *ATAF1* showed improved tolerance to dehydration, ABA, salinity and oxidative stresses in *Arabidopsis* (Wu et al., 2009). Overexpression of *OsNAC10, OsNAC6* and *OsNAC5* genes significantly promoted the survival of the rice plants under high salinity and water deficit (Hu et al., 2006; Song et al., 2011).

NAC family members normally contain a highly conserved DNA-binding domain at the N-terminal composed of about 150–160 amino acid (aa) which is further subdivided into 5 (A–E) subdomains, but C-terminal is highly variable and consists of the transcriptional regulatory domain (TRD), which can either function as a transcriptional promoter or repressor (Kikuchi et al., 2000; Ooka et al., 2003). A special category of NAC members of NTL (NAC with transmembrane motif 1-like) has α-helical transmembrane motifs (TMs) at their C-terminal (Kim et al., 2010; Kim et al., 2007). These features facilitate inactivation or repression of various downstream genes controlling multiple cellular or molecular processes.

Because of the critical regulatory functions of *NAC* genes in plant responses to different stresses, the NAC gene family has been extensively studied in *Arabidopsis thaliana* and some other important plant species. To date, however, no genome-wide characterization of the NAC family has been conducted in radish. Therefore, it is important to comprehensively investigate the NAC TF family in radish to utilize them to develop stress tolerance cultivars in radish and other related root vegetables. The genome sequences of radish have been recently sequenced by Jeong et al. (2010), Kitashiba et al. (2014) and Mitsui et al. (2015) which allows for the in-depth analysis of the NAC TF family. In this study, 172 *RsNAC* genes were identified from the radish genome database and classified according to previously reported *Arabidopsis* and *B. rapa NAC* gene families. In addition, phylogenetic analysis, chromosome location and membrane-bound protein identification analysis were performed. Moreover, expression patterns of *RsNAC* genes in response to HMs, salt, heat, drought and ABA stresses were further investigated using qRT-PCR. These results will improve our understanding of the NAC gene family in radish, as well as contribute to screening of additional elite genes for future functional investigation of *RsNACs* under abiotic stresses.

## Materials and Methods

### Database search and detection of NAC transcription factors

The protein and nucleotide sequence datasets were obtained from the radish genome database for the detection of the RsNAC gene family (Mitsui et al., 2015). Radish protein sequences were retrieved using Hidden Markov Model profile (PF02365) generated from Pfam (Punta et al., 2012) with a cut-off *E*-value of 1e−3. To confirm the presence of conserved NAC domains, multiple alignment of full-length RsNAC protein sequences was carried out using the clustalW program with default pairwise and multiple alignment parameters. The data for *Arabidopsis, B. rapa* and rice NACs sequences were retrieved from the *Arabidopsis* Information Resource (Huala et al., 2001), Brassica Database (Cheng et al., 2011) and the Rice Genome Annotation Project (Kawahara et al., 2013), respectively. The sequences of all NAC TFs in other representative species were retrieved from the plant TF database (Jin et al., 2014). Additionally, TMHMM Server v. 2.0 (Krogh et al., 2001) was employed in discovering membrane-bound RsNAC proteins.

### Phylogenetic analysis of RsNAC protein domains

Phylogenetic analysis was done using RsNAC domain sequences with previously grouped NAC domains from *Arabidopsis* and rice as reference sequences of eudicots and monocots, respectively (Abrouk et al., 2010). The phylogenetic tree was generated using MEGA6 program by the neighbor-joining method with 1,000 bootstrap replicates (Tamura et al., 2013).

### Protein characterization and RsNAC sequence analyses

The Protparam program (Gasteiger et al., 2005) using ProtParam tool was employed to determine the molecular weight (MW) and isoelectric points (pI) using default settings. The online MEME program (Bailey et al., 2009) was used to search conserved motifs. The analysis was performed with default parameters except the following: number of repetitions, any; maximum number of motifs, 15; and optimum width of the motif, ≥50. The information for each *RsNAC* exon/intron organization was determined by mapping the CDS to DNA sequences using the Gene Structure Display Server 2.0 GSDS (Hu et al., 2014). To determine *Arabidopsis* orthologs, radish NAC proteins were used in BLASTP search against the *Arabidopsis* Information Resource, TAIR10 release (Huala et al., 2001). Reciprocal best BLAST hit blasting approach(Moreno-Hagelsieb & Latimer, 2008) was used and best hits were considered when bit-score of the compared proteins was more than 200, the sequence coverage value more than 50% and expected value *E*- value of ≤1e–3.

### Chromosomal location of *RsNAC* genes

The sequences of *RsNAC* retrieved from the genomic sequences of the scaffolds were anchored to the integrated genetic map published byJeong et al. (2010). All the acquired potential *RsNAC* genes were searched against radish the chromosome sequence database Jeong et al. (2010) using BLASTN program. The sequences with a similarity of ≥99% and length coverage difference ≤5 base pairs (bp) were regarded as similar genes (Wang et al., 2015b). Subsequently, all the similar *RsNAC* gene locations (megabase, Mb) on the genetic map were annotated according to their corresponding pseudomolecules. All anchored genes were located on their specific genetic map against their location parameters using MapInspect software (http://www.plantbreeding.wur.nl/UK/software_mapinspect.html). Tandem repeats located within four predicted genes or within 50 kb from each other were identified manually and marked on the radish genetic map (Cannon et al., 2004).

### Expression analysis of *RsNAC* genes based on the RNA-Seq data

The normalized RNA-Seq data were retrieved from Nodai Radish Genome Database (Mitsui et al., 2015), and then used for the expression profiling of the *RsNACs* in two tissues (roots and leaves) and six developmental stages (7, 14, 20, 40, 60 and 90 days after sowing, DAS) represented by RPKM (Reads Per Kilobase of transcript per Million mapped reads) value.

### Plant growth, abiotic stress treatments and expression analysis

The ‘NAU-YH’ genotype seedlings were germinated on a wet filter paper in darkness for three days then transferred to the pots containing soil and peat media (1:1) and cultured in the growth chamber under 16-h (25 °C) and 8-h (18 °C) light and dark condition, respectively. Thirty days after transplanting, seedlings were subjected to the different treatments. For heat and salt treatments, seedlings were exposed to 42 °C and 200 mM NaCl conditions, respectively. For HMs treatments, seedlings were exposed to 20 mg L^−1^ CdCl_2_ ⋅2.5H_2_O and 100 mg L^−1^ Pb (NO_3_)_2_, respectively. For drought treatment, seedlings were treated with 20% polyethylene glycol 6000 (PEG) while 100 µM ABA was sprayed to the seedlings to induce ABA response. The samples from all the treatments were harvested after 24 h and frozen immediately in liquid nitrogen for RNA extraction. Total RNAs from triplicate samples were isolated using Trizol (Invitrogen, Carlsbad, CA, USA) according to manufacturer’s protocol and then digested with RNase-free DNase. The quality of total RNA was determined by agarose gel electrophoresis *.* The total RNA (2 µg) was used to synthesize the first-strand cDNA using PrimeScript™ RT Reagent Kit (TaKaRa, Dalian, Liaoning Sheng, China), and the obtained cDNA products were further diluted 1:9 with nuclease-free water for RT-qPCR. The transcript levels of 21 randomly selected *RsNAC* genes were investigated with RT-qPCR according to the method reported by Xu et al. (2013). The radish *Actin* gene was used as the internal control. The Beacon Designer 7.7 was used to generate gene-specific primers from the non-conserved region of NAC sequences (Table S1).

## Results

### Identification of the *RsNAC* genes and comparative analysis in radish

In the determination of *NAC* genes in radish, a total of 181 *NAC* genes were obtained after searching the entire radish genome by the HMMER software. Furthermore, SMART and Pfam programs (Letunic, Doerks & Bork, 2012; Punta et al., 2012) were used to further screen the presence of the NAM domain in all the RsNAC sequences with a cut-off *E*-value of 1e–3. Finally, 172 RsNAC proteins were selected for subsequent analysis after exclusion of nine members with either no N-terminal NAM domain or with an *E*-value of >1e–3. For nomenclature, the family designation (NAC) was preceded by prefix ‘Rs’ for radish, and then followed by a number based on their generic order. The comprehensive list of RsNAC proteins analyzed in this study, the annotation of each sequence and their preatomic characteristics are shown in Table S2. The number of aa in RsNAC proteins ranged from 143 (RsNAC143) to 652 (RsNAC011) residues resulting in an average of ∼336 aa, whereas the pI ranged from 4.44 (RsNAC0730) to 9.63 (RsNAC173) with an average of ∼6.6. The results indicated that different *NACs* genes might function in diverse microenvironments (Kiraga et al., 2007). The RsNAC domains were aligned and observed by GeneDoc program. The RsNAC proteins possess an N-terminal NAM domain which is highly conserved compared to the diverse C-terminal (Figs. S1A–S1C). Moreover, the N-terminal NAM domains of RsNAC proteins were grouped into five distinct clusters (A–E) which were similar to that in *A. thaliana* and *C*. *sativus* (Tran et al., 2004; Zhang et al., 2017). However, among the 172 RsNACs, only the RsNAC155 lack the conserved subdomains A, B and C, while RsNAC161 and RsNAC127 do not contain subdomain A and C, respectively. Additionally, several proteins (RsNAC172, RsNAC171, RsNAC098, RsNAC100, RsNAC101, RsNAC156 and RsNAC143) lack subdomain E (Fig. S1C).

**Figure 1:**
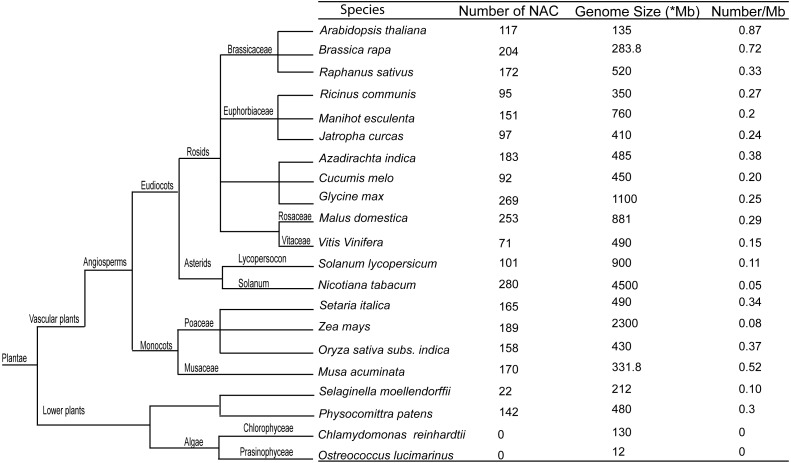
Comparative genomic analysis of NAC transcription factors of radish with other species. There were 17 vascular plants and four lower plants. *Megabases.

For comparative genomic analyses, we obtained NAC protein coding sequences for 21 representative plant species (Fig. 1), resulting to 2931 NAC TFs. Interestingly, all the vascular plants had *NAC* genes while in lower plants, two algae representatives had none. The phenomenon suggests that the NAC proteins might have expanded after the evolutionary split of the vascular plants from the lower plant species, and further indicate that this family is vascular plant-specific based on the missing NACs in algae. In conclusion, based on this study, the number of NAC in radish ranked among the members with high NAC representation in their genome. According to the density of NACs in the respective genomes, radish (0.33) ranged among the species with a high number of NAC in our analyses (Fig. 1). It was also noted that the densities of NAC TFs in most of the higher plant genomes were greater than those in all the lower plant genomes, revealing that NAC TFs increased considerably with the evolution of higher plants.

### Phylogenetic analysis of NACs in radish

**Figure 2:**
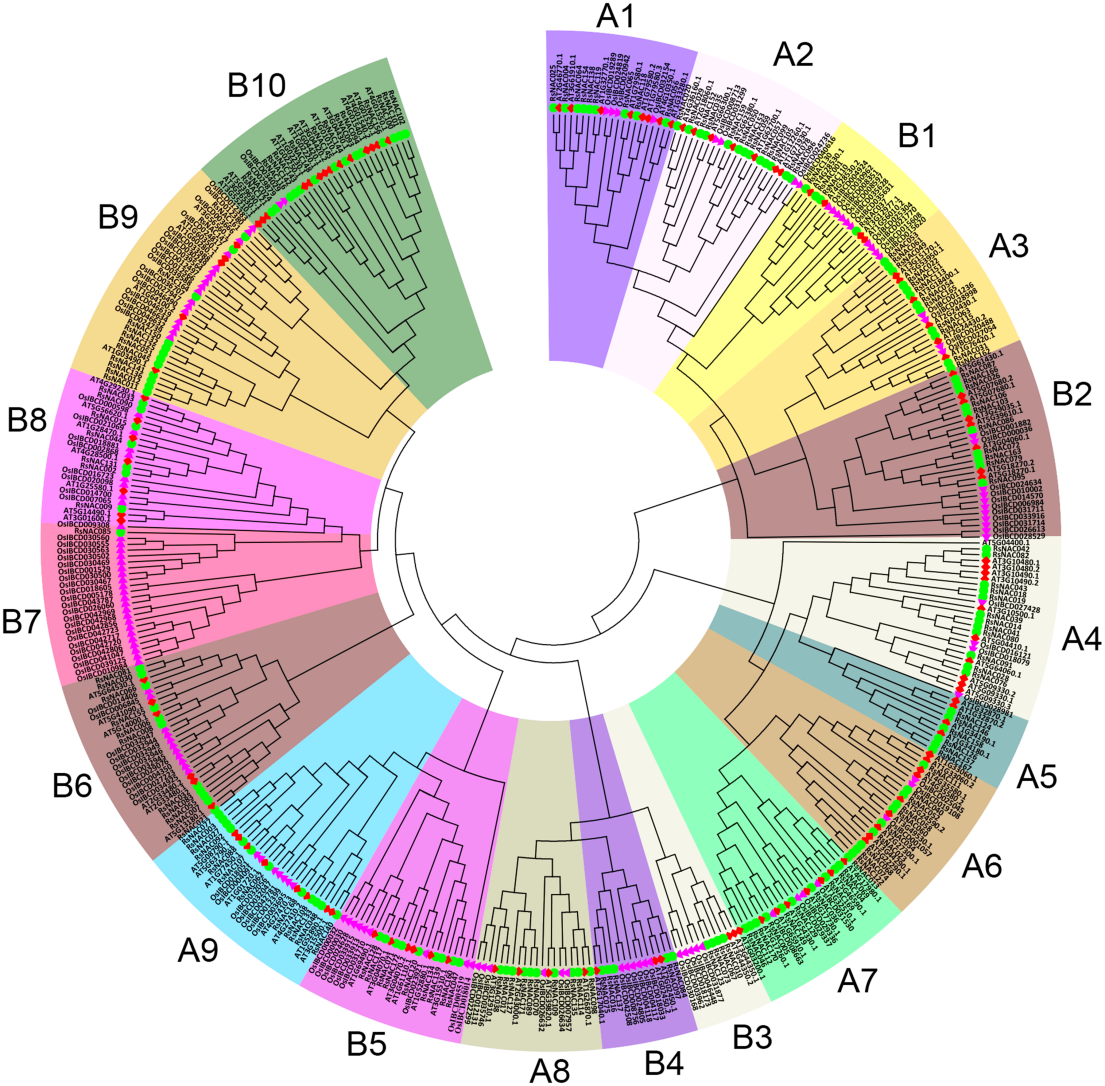
Phylogenetic tree among the NAC TFs of radish, *Arabidopsis*, and rice. Multiple sequence alignment of full-length NAC proteins was done using ClustalW and the phylogenetic tree was constructed using MEGA6 by the neighbor-joining method with bootstrap replicates, the tree was divided into two major groups (A and B). Group A was further subdivided in into nine phylogenetic subgroups designated A1–A9. Phylogenetic subgroups in-group B were designated as B1 to B10. The different sub-families are marked with different colored backgrounds. The colored shapes next to names represent radish (green circles), Red hexagons (*Arabidopsis*) and purple hexagons (rice).

To gain further insights into the evolutionary relationship among NAC members from different plants, the NACs from radish were aligned against those in *Arabidopsis* and rice, and to plot an unrooted phylogenetic tree using MEGA 6 software (Fig. 2, Table S3). The RsNACs could be broadly categorized into two groups (group A and group B, respectively) based on their preferential homology to *Arabidopsis* and rice. As a result, group A and B showed high homology to *Arabidopsis* and rice, respectively. Additionally, 9 and 10 subgroups were obtained from group A (A1–A9) and B (B1–B10) in the phylogenetic analysis of the full-length NAC proteins of *Arabidopsis*, rice and radish based on similarity in NAC domain structures. Phylogenetic analysis revealed that most RsNAC members were closer to eudicot (AtNACs) than monocot (OsNACs) and preferentially clustered in group A. For example based on outer clades with a bootstrap value of >50, 12 RsNACs clustered with 10 AtNACs in A4 while 23 OsNACs clustered with only one RsNAC member (RsNAC085) in subgroup B7 (Fig. 2, Table S3).

### Motif and gene structure analysis of the *R*s*NAC* genes

**Figure 3:**
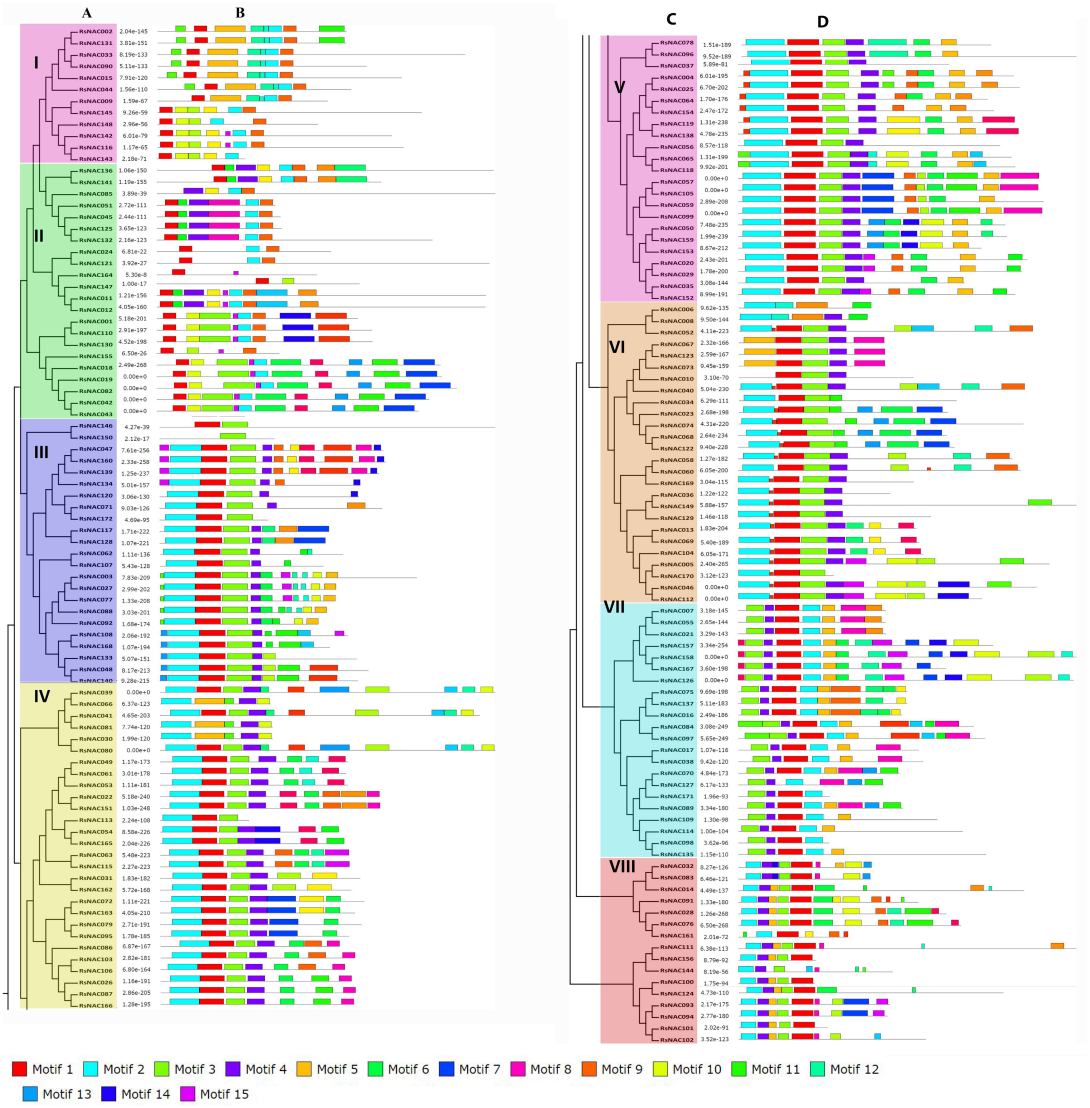
Phylogenetic classification and motif analysis of NAC transcription factors of radish. Multiple sequence alignment of 172 full-length RsNAC proteins was done using ClustalW, and the phylogenetic tree was mapped using MEGA6 by the neighbor-joining method with 1,000 bootstrap replicates. MEME analysis explored conserved motifs of the RsNAC proteins. Gray lines illustrate the non-conserved sequences and each motif is showed by the colored boxes described at the bottom. The different color background indicated different groups. (A and C) multiple alignment, (B and D) conserved motifs.

**Figure 4:**
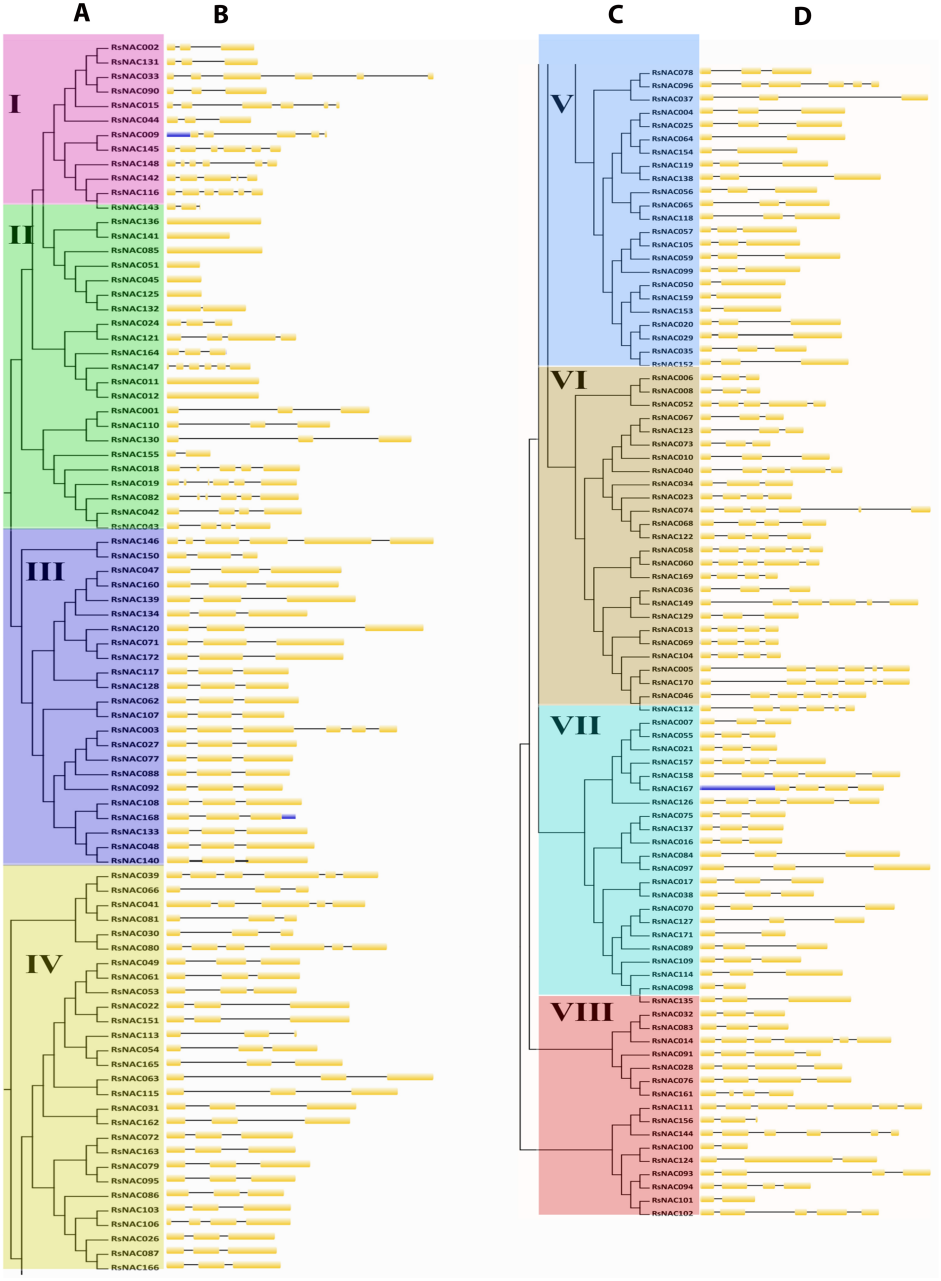
Phylogenetic tree and Exon/Intron structure of 172 *RsNAC* genes. Yellow boxes and black lines represent exons and introns, respectively, while the blue boxes represent the untranslated regions. (A and C) multiple alignment, (B and D) gene structure composition.

To explore the relationships among the *RsNAC* genes and predict their potential functions, the RsNAC proteins clustered into 9 subgroups based on the phylogenetic tree (Figs. 3A and 3C), named I to VIII. The gene number of each group ranged from 12 (subgroup I) to 28 (subgroup IV). RsNAC proteins belonging to a common cluster in the phylogenetic group (Figs. 3A and 3C) indicated a common motif distribution (Figs. 3B and 3D), suggesting functional similarities among members within the same subset. Fifteen (1–15)conserved motifs were analyzed in the RsNAC proteins (Figs. 3B and 3D). Notably, the majority of the discovered motifs were located in the N-terminal region of the NAC domain which could be an indication that these motifs are critical for the functioning of NACs. For example, subgroup I mainly contained six motifs (motifs 1, 2, 4, 5, 8 and 9), whereas subgroup II comprised of seven motifs (motifs 1, 2, 3, 4, 5, 7 and 13), which suggests that the RsNACs with similar functions tended to cluster into the same subgroups. Additionally, the structural features of the intron/exon distribution among *RsNAC* genes were analyzed based on their phylogenetic similarities (Figs. 4A–4D, Table S2). Exon/intron analysis depicted that introns distribution in *RsNAC* genes is diverse and varied from 0 to 6. Additionally, a total of 164 (∼95%) *RsNAC* genes had introns, while 8 (∼5%) *RsNACs* contained no intron (Figs. 4C and 4D). Among them, one *RsNAC* (*RsNAC111*) had six introns, two sets of 13 *RsNAC* members had five or four introns each and 109 *RsNAC* genes had two introns each. Particularly, the *RsNACs* with a maximum number of introns were located in subgroup VI with seven members having five introns, while the *RsNACs* without intron were located in subgroup I. These findings indicated a minimum diversity in gene structure among *RsNAC* genes belonging to a common group.

### Membrane-associated RsNAC subfamily

**Figure 5:**
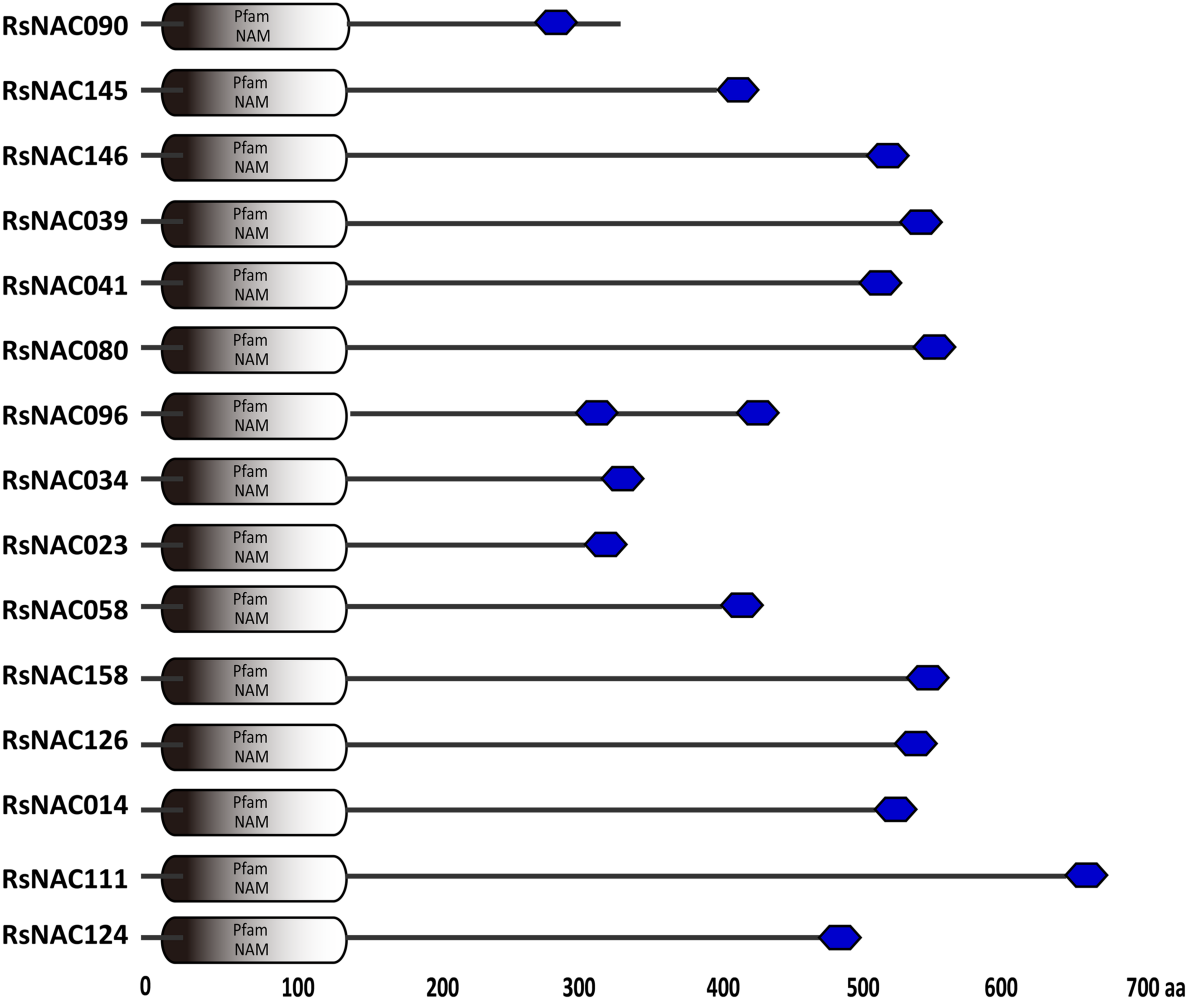
Membrane-associated RsNAC subfamily. Protein structure of membrane-bound NAC TFs poses indicating a conserved NAM domain located at the N-terminal of the proteins, TMs (transmembrane motifs) located at the N-terminal as shown by the blue hexagon.

Fifteen RsNAC proteins (∼8.72%) were identified as membrane-associated RsNACs proteins (Fig. 5, Table 1). Classification of NTL proteins among the subgroups was discrete. *Arabidopsis* orthologues analysis indicated that 8 RsNAC NTLs were orthologs to previously characterized NTL from *Arabidopsis* (Table 1). The subgroups IV, VI and VIII contained three NTL proteins, while groups I and VII contained 2 NTLs, indicating that the functions of radish NTLs were complex.

**Table 1:** Description of the predicted membrane-bound NAC in radish.

Protein	Length (aa^a^)	Tm^b^ helix	At^c^ orthologue locus	At locus description
RsNAC090	339	281–303	AT4G28530.1	ANAC074
RsNAC145	428	410–428	AT5G14490.1	ANAC085
RsNAC146	546	517–539	AT3G10480.1	ANAC050
RsNAC039	568	540–562	AT3G15500.1	ANAC055
RsNAC041	535	510–532	AT5G64530.1	ANAC104
RsNAC080	570	545–567	AT5G64530.1	ANAC104
RsNAC096	444	309–331	AT1G71930.1	ANAC030
RsNAC096	444	425–442	AT1G71930.1	ANAC030
RsNAC034	349	323–345	AT3G49530.1	ANAC062 (NTL6)
RsNAC023	335	317–335	AT2G27300.1	ANAC040 (NTL8)
RsNAC058	440	408–430	AT5G22290.1	ANAC089 (NTL17)
RsNAC158	573	541–563	AT1G34190.1	ANAC017 (NTL7)
RsNAC126	565	534–556	AT1G34190.1	ANAC017 (NTL7)
RsNAC014	547	521–543	AT5G04410.1	ANAC078 (NTL11)
RsNAC111	652	612–634	AT1G33060.1	ANAC014 (NTL2)
RsNAC124	509	480–502	AT1G32870.1	ANAC013 (NTL1)

**Notes.**

a Amino acid.

b Transmembrane segments predicted using online TMHMM server 2.0.

c *Arabidopsis thaliana*.

### Chromosomal distribution of *RsNAC* genes in the radish genome

**Figure 6:**
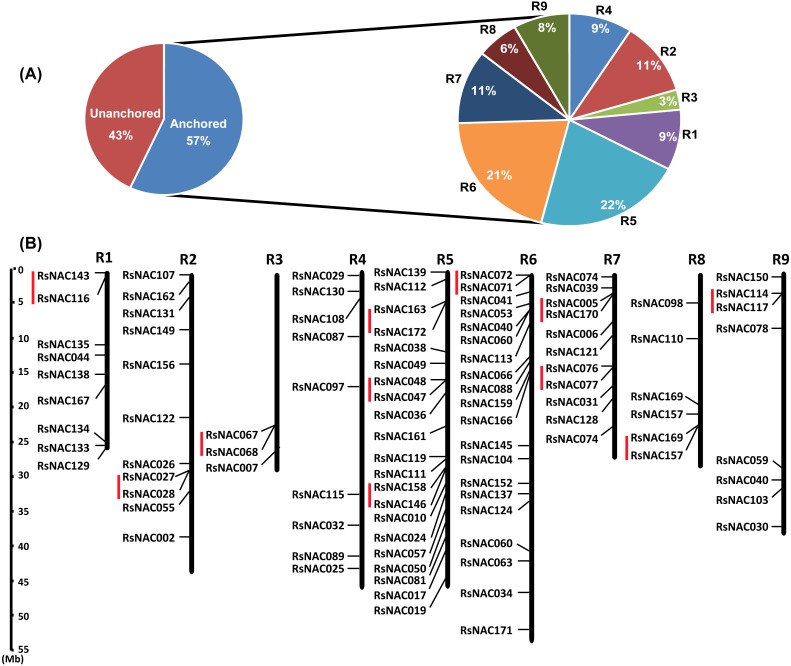
Chromosomal distribution of *RsNAC* genes on the genetic map. (A) A pie chart is indicating the percentage of the anchored and unanchored *RsNAC* genes and their abundant distribution on each radish chromosome with indication of percentages of *RsNACs* located on each chromosome. (B) Graphical representation of locations for putative *RsNAC* genes on each radish chromosome. The vertical red lines on the right of each chromosome indicate tandem duplicated genes. R1–R9 indicates the chromosome numbers. The scale is in megabase.

A total of 98 (∼57%) *RsNAC* genes were mapped onto 9 chromosomes (R1–R9) (Fig. 6, Table S4). For distribution and density of the *RsNAC* genes, R5 contained the maximum frequency (∼22%) of *RsNAC* genes followed by R6 (∼21%), while R3 had the lowest frequencies of *RsNAC* genes ∼3% (Fig. 6A). Thirteen *RsNAC* gene clusters containing 26 tandemly duplicated genes were identified in all chromosomes except chromosome R4 (Fig. 6B). Most tandem duplications were located on chromosome R5 with three gene clusters containing two genes each. The phenomenon indicated that some of the *RsNAC* genes have one or more paralogs, which could have been generated by whole-genome duplication (WGD) in radish. In general, majority of the *RsNAC* were located in the distal regions of the nine radish chromosomes while a few were found in proximal regions. Additionally, the *RsNAC* gene density in each chromosome ranged from 0.10 /Mb (R3) to 0.45 /Mb (R5). This indicated that the *RsNAC* genes were significantly non-random and unevenly distributed in the nine radish chromosomes.

### RNA-Seq-based expression analysis of *RsNAC* genes

For further investigation on the roles of *RsNAC* gene, the RNA-Seq based analysis was utilized to explore the expression patterns of the 172 *RsNACs* in roots and leaves at six different developmental stages (Fig. S2A–S2D, Table S5). As shown in Figs. S2A and S2C, there was no detectable expression of four *RsNACs* genes (*RsNAC002*, *131*, *039* and *008*). Moreover, tissue-specific expression patterns were observed in several *NAC* genes. These genes could be subdivided into three expression categories, which contained genes preferentially expressed in roots, leaves and both. For example, *RsNAC002* (group I), *RsNAC160* (group III), R*sNAC165* (group IV), *RsNAC069* (group V), *RsNAC008* (group VI), *RsNAC126* (group VII), and *RsNAC124* (group VIII) were preferentially expressed in roots, while *RsNAC097* and *RsNAC017* (group VII) showed biased expression in leaves. However, some *RsNACs* exhibited multiple expressions in more than one organ and developmental stages. For example, *RsNAC001*, *RsNAC027*, *RsNAC080* and *RsNAC040* (group VI), *RsNAC007* (group VII) and *RsNAC028* (group VIII) were detected in roots and leaves at the six stages of development.

### *RsNAC* genes expression profiling under various abiotic stresses and phytohormone treatment

**Figure 7:**
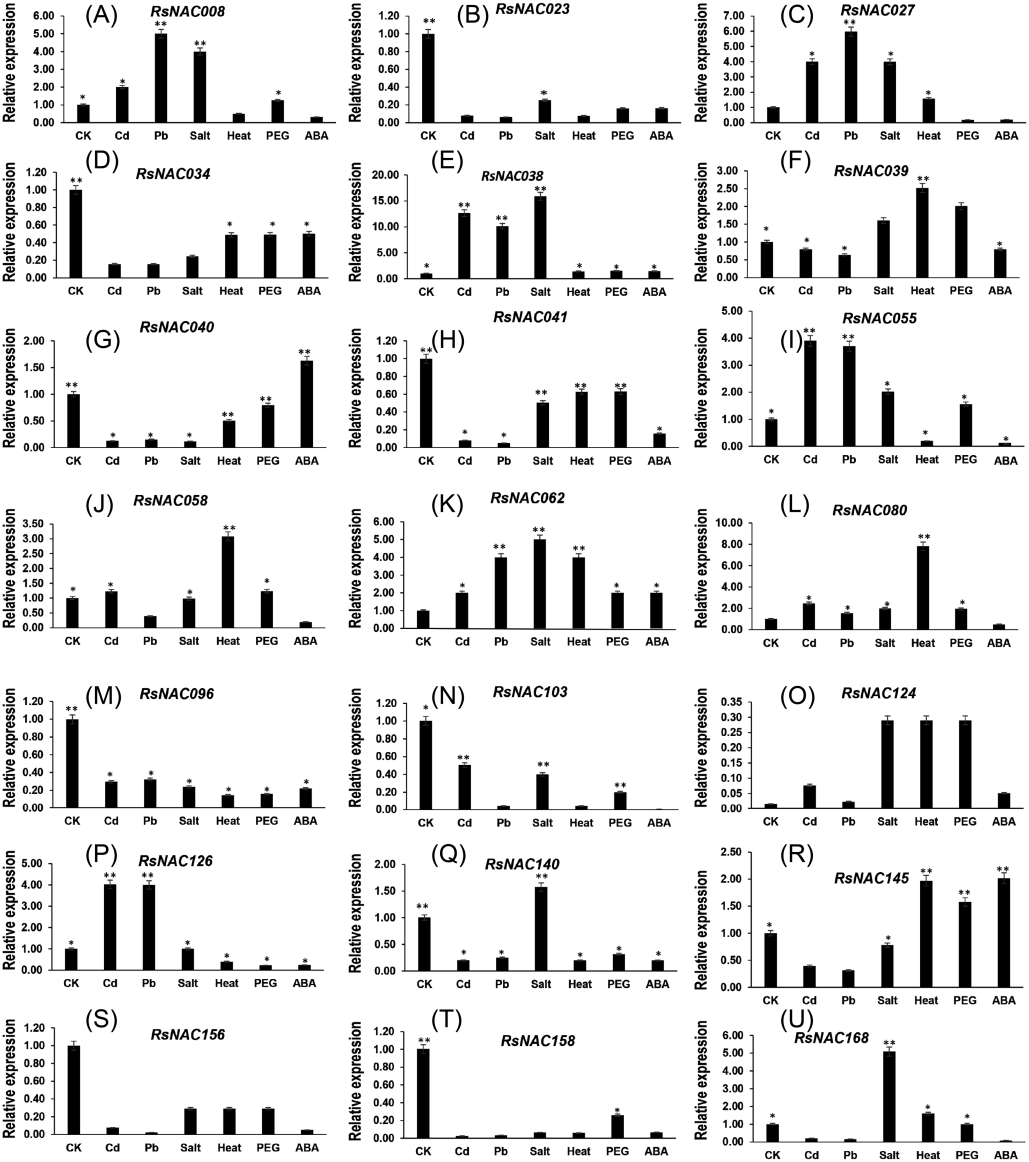
Expression patterns of *RsNAC* genes under abiotic stresses treatments. RT-qPCR was used to analyze the expression profiles of 21 *RsNAC* genes under cadmium, lead, heat, salt, PEG, and ABA stress. CK is an untreated seedling. The *Actin* gene was used as an internal control. (A–U) represents expression patterns of the 21 RNAC genes in response to abiotic stresses, respectively. Error bars were obtained from three technical replicates. Asterisks indicate the genes significantly up-regulated or suppressed under abiotic stresses by *t*-test (* *P* < 0.05, ** *P* < 0.01).

To explore the expression patterns of *R*s*NAC* genes under various abiotic stresses and hormone treatments, 21 representative genes were identified for expression analysis under Cd, Pb, salt, PEG and ABA treatments. The results showed differential expression patterns of these genes under one or more treatments(Fig. 7, Table S6). Some genes (*RsNAC027*, *RsNAC038* and *RsNAC062*) showed stable up-regulation under Cd, Pb and salt treatments. However, the other genes showed a weak expression level under Cd and Pb stress. Most *RsNAC* genes were preferentially down-regulated under HM stress whereas Pb stress up-regulated only a few of them, including *RsNAC038*, *RsNAC008* and *RsNAC062* (Fig. 7).

## Discussion

### Characterization of *RsNAC* genes

NAC TFs are one of the largest plant-specific transcriptional regulators that play critical roles in numerous stress and developmental processes. However, there is still no detailed information available on NAC family in radish. To further explore the NAC information in radish, 172 putative *RsNAC* genes were identified from the radish genome. Remarkably, the number of the RsNAC members ranges among the largest NAC families. This finding is probably due to the selective pressure conferred by environmental cues to facilitate the growth and development of plants (Hughes & Friedman, 2003). In comparative genomic analysis among different plant species, *N. tabacum* was revealed harboring the highest numbers of NAC TFs (280). However, their NAC protein density (*N. tabacum*, 0.055) was lower than those in radish because of their large genome sizes. Additionally, stress responses of several *RsNAC* genes were detected based on their corresponding *Arabidopsis* homolog, which could be of benefit for further studies in functional characterization of *NACs* genes in root vegetables.

According to multiple sequence alignment, all the identified RsNACs contain the expected NAM domain (Pfam PF02365) with approximately 160 aa apportioned into five distinct subdomains (A–E). However, several RsNACs have no typical NAC domain pattern. For example, RsNAC155 lacks the conserved domain A, B, C and D, while other 12 RsNACs lacked one or two sub-domains. Such variant NAC members are termed as NAC-like proteins as described in potato and rice NACs (Nuruzzaman et al., 2010; Singh et al., 2013). The C-terminal region of the RsNACs domains indicate a highly distinct pattern involved in biotic and abiotic stress processes (Mao et al., 2012). Previous studies revealed the involvement of NAC members in protein-binding activity which performs major functions in transcription regulation (Jensen et al., 2010; Nuruzzaman et al., 2010). For phylogenetic analysis, monocots specific phylogenetic clade was identified with only one RsNAC member. The results suggest the existence of evolutionary differences between NACs from monocots and dicots and a lineage-specific expansion after splitting from their common ancestor. Additionally, the majority of the monocots and dicots with diverse functions belonged to common clades indicating that greater expansion of the NAC family could have happened slightly before the splitting of monocots and dicots (Pereira-Santana et al., 2015). Based on the results of comparative genomic analysis, it was possible to predict the functions of several *RsNAC* genes in response to abiotic stress according to their *Arabidopsis* homologs*,* which could also be potentially utilized for further functional studies. It was found that *ANAC019*, *ANAC055* and *ANAC07* which are orthologs to *RsNAC168*, *RsNAC140* and *RsNAC105,* respectively, have been implicated in positively responding to dehydration in *Arabidopsis* (Hu et al., 2006; Tran et al., 2004).

In this study, all the identified RsNAC NTLs proteins contained single TM at the C-terminal of their conserved domain except RsNAC096 with two TMs, which is similar to two soybean NAC proteins (*GmNAC136* and *GmNAC013*) where each of them contained a pair of TMs (Le et al., 2011). Recently, NTLs were demonstrated to have critical functions in response to multiple stresses (Kim et al., 2007; Lee et al., 2014). Moreover, NTLs facilitates an efficient mechanism for gene regulation which is a survival feature that allows for timely responses to environmental cues (Kim et al., 2007). In *Arabidopsis*, it has been reported that four NTLs (*At4g01540*, *At2g27300*, *At4g35580* and *At3g49530*) are involved in abiotic stress tolerance, which are membrane-related proteases located in the endoplasmic reticulum that initiates the activities of TFs in response to stresses (Chen, Slabaugh & Brandizzi, 2008; Kim et al., 2008; Seo, Kim & Park, 2008). It was found that the radish NTLs (RsNAC034, RsNAC023, RsNAC058, RsNAC158, RsNAC126, RsNAC014, RsNAC111 and RsNAC124) showed close phylogenetic relationship with AT3G49530.1, AT2G27300.1, AT5G22290.1, AT1G34190.1, AT1G34190.1, AT5G04410.1, AT1G33060.1 and AT1G32870.1, respectively. In *Arabidopsis,* these NAC were also identified as NAC NTLs (Kim et al., 2007). Thus, it is expected that some identified RsNAC NTLs could function critically in the adjustment of stress-responsive genes as a regulatory mechanism for the survival of radish under adverse growth conditions.

### Chromosomal distribution and expression profiling of *RsNAC* genes

The vast number of genes in NAC family in radish confirms the occurrence of chromosome duplication events during evolution. In angiosperms, several genome duplications have occurred resulting in gene family enlargement (Cannon et al., 2004), this phenomenon occurred in radish about 5.1–8.4 and 12.8–21.4 million years ago (Shen et al., 2013). Genome duplication provides a critical evolutionary promoter in angiosperm genome enlargement (Van de Peer et al., 2009). Additionally, most studies have indicated that WGD is a key factor in angiosperm biology. During genome duplication processes, redundant genes are lost (fractionation), preserved, or fixed to perform a diverse task compared with the original genes. Therefore, gene family size could vary significantly among species due to WGD events that took place during their evolution (Abrouk et al., 2010; Cenci et al., 2014). In several TFs, genome duplication has been reported in plants including MYB and WRKY, which is the same in NAC (Karanja et al., 2017; Liu et al., 2014; Nuruzzaman et al., 2010). In this study, eleven clusters representing probable tandem duplication formed by *RsNAC* genes in the genome were identified to elucidate the mechanism behind a large number of the NAC family members in radish. In *Arabidopsis*, many gene families have enlarged due to the retention of original genes after WGD and transposition (Freeling et al., 2008; Rizzon, Ponger & Gaut, 2006). Each of these gene family expansions forms paralogs that potentially lead to genetic redundancy in addition to differential growth among different families (Pereira-Santana et al., 2015). This phenomenon could contribute to the expansion of the RsNAC TFs in radish, similar to *B. rapa* and *M. domestica* (Liu et al., 2014; Su et al., 2013). Tandem duplication also indicate the presence of *redundant* genes which according to existing theory cause loss or mutation of duplicate genes despite retention of several genes after WGD by functionalization (Lynch & Force, 2000).

In the present study, the publicly available RNA-Seq data was utilized in conducting *in silico* expression analysis of *RsNAC* genes. Transcript abundance of *NAC* genes in roots and leaves reveal a spatial, temporal and ubiquitous pattern of *RsNAC* genes of which *RsNAC168* was preferentially expressed in leaves, which was similar to its corresponding orthologs in *Arabidopsis* (*ANAC019*) expressed in leaves for regulating drought and salinity responses (Jensen et al., 2010). Additionally, *ANAC019* and *ANAC055* (orthologs to *RsNAC168* and *RsNAC140*, respectively) are involved in both ABA and JA-mediated regulation (Jensen et al., 2010). Notably, most expressed genes were responsive in more than one tissue and developmental stage, spatio-temporal *expression pattern* of the *RsNACs* could provide the specific usage of *RsNACs* in a transcriptional modification in any given tissue, while ubiquitously regulated *RsNACs* could control the transcription of a wide range genes. In rice, *OsNAC10* is widely expressed in roots and flowers tissues in response to dehydration and salinization stresses (Jeong et al., 2010).

### Differential expression of *RsNAC* genes under various abiotic stresses

Unfavorable environmental cues such as HMs, salinization, high temperature and dehydration could result in permanent and deleterious effects on the optimal survival of plants. In this study, the response of *RsNAC* genes to all the stress treatments indicate their integral role in regulating survival processes associated with stress response and signaling in radish and other related species. According to the previous report, NAC TFs were reported to coordinate stress by initiating stress-responsive genes (Jensen et al., 2010). Several *RsNACs* (including *RsNAC008, RsNAC027, RsNAC038*) were significantly upregulated in response to salt. In *Arabidopsis*, overexpression of *ANAC072, ANAC055* (*RsNAC039, RsNAC140*) and *ANAC019* (*RsNAC168*) showed improved stress tolerance under salt conditions than the wild-type (Hu et al., 2006; Jensen et al., 2010). The finding could suggest that up-regulating of these genes may have resulted in improved salt tolerance in radish, however, *NAC* genes were found to negatively control drought responses by up-regulating the expression of integral drought- responsive genes(Wang et al., 2013b). Some genes, such as *RsNAC030*, *RsNAC041* and *RsNAC145*, showed diverse responses under different stresses, indicating that these may function as links between the various transduction pathways (Liu et al., 2014).

In recent years, NTLs have been extensively proven to play critical roles in cell division as well as abiotic stress responses (Lee et al., 2014). In selective breeding, genes with multiple stress responses possess high potential of stress tolerance manipulation in plants. However, an occasional overexpression of *NAC* genes may result in antagonistic responses such as delayed flowering and dwarfing in transgenic plants (Liu et al., 2011). Overexpression of A*NAC019* and *ANAC055* in *Arabidopsis* resulted in increased drought tolerance but also increased susceptibility to *B*. *cinerea* (Bu et al., 2008). Field evaluation and prudent choice of a suitable promoter can reduce the negative effects in the over-expression of some *NAC* genes (Shao, Wang & Tang, 2015). In this study, nine NTLs (*RsNAC23*, *RsNAC34*, *RsNAC39, RsNAC041*, *RsNAC058*, *RsNAC080*, *RsNAC096*, *RsNAC124* and *RsNAC145*) were among the 21 responsive *RsNAC* genes that responded to multiple abiotic stresses. Among them, *RsNAC023* and *RsNAC080* responded to all stress treatments other than ABA, while *RsNAC145* responded to salt, heat and drought only. Evidently, functional characterization of these genes could be of great interest in unraveling their potential for the development of radish cultivars tolerant to multiple abiotic stresses. Over-expression techniques will provide valuable insights into understanding the regulatory functions of NAC members in radish in response to abiotic stresses. Significantly, NTLs responded differently to various stress treatments, *RsNAC058* was upregulated under Cd, heat and drought, but downregulated under Pb and ABA it was. The result implied that the *NTL* genes might be playing critical roles in plant response to abiotic stresses.

## Conclusion

Characterization of NAC TF gene family in radish was performed with particular emphasis on their responses to abiotic stresses. In total, 172 *RsNAC* genes were identified and characterized in radish. The conserved motif, gene structure, chromosomal distribution and expression profiles of the *RsNAC* genes in response to abiotic stresses were analyzed. The expression profiles of *RsNAC* genes under Cd, Pb, heat, salt, PEG and ABA stress displayed differential expression pattern in response to one or more stresses. In this study, it was observed that several *RsNAC* genes could play key roles in conferring tolerance to different abiotic stresses. The findings could provide fundamental information for further functional studies of the *NAC* genes by unraveling potential *NAC* genes in the radish, and facilitate manipulating multiple stress tolerances in the radish and related root vegetables. The result could also facilitate developing agronomically superior traits for radish including enhanced tolerance to abiotic stresses.

##  Supplemental Information

10.7717/peerj.4172/supp-1 Figure S1A Description of RsNAC sequences of sub-domain A and B Multiple alignment of full-length RsNAC protein sequences was carried out using the clustalW program with default pairwise and multiple alignment parameters.

10.7717/peerj.4172/supp-2 Figure S1B Description of RsNACs sequences of sub-domain C and D Multiple alignment of full-length RsNAC protein sequences was carried out using the clustalW program with default pairwise and multiple alignment parameters.

10.7717/peerj.4172/supp-3 Figure S1C Description of RsNAC sub-domain E sequences Multiple alignment of full-length RsNAC protein sequences was carried out using the clustalW program with default pairwise and multiple alignment parameters.

10.7717/peerj.4172/supp-4 Figure S2 Heat map showing AP2, RAV and soloist members expression pattern in roots and leaves tissues of radish at various developmental stages The color scales for fold-change values are shown at the bottom such as elevated (red) and suppressed (green) genes. Several genes were not expressed (black) in any tissue.

10.7717/peerj.4172/supp-5 Table S1 List of 21 primers used for expression analysis Gene-specific primers generate from the non-conserved region of NAC sequences using Beacon Designer 7.7.

10.7717/peerj.4172/supp-6 Table S2 Characteristic features of NAC Transcription factor gene family identified in *R. sativus* ^a^Transmembrane motif predicted using the TMHMM server
^a^Amino acid ^c^Molecular weight
^d^Isoelectric points
^e^ *Arabidopsis thaliana*

10.7717/peerj.4172/supp-7 Table S3 Detailed information of the Phylogenetic tree among the NAC TFs of radish, *Arabidopsis* and rice Clustering of groups A and B based on the similarity in NAC domain structures, group A contain nine subgroups (A1–A9) mostly related to eudicot while group B is composed of ten subgroups (B1–B10) mostly related to monocot.

10.7717/peerj.4172/supp-8 Table S4 The information of genetic map distribution of *RsNAC* genes *Megabase

10.7717/peerj.4172/supp-9 Table S5 The RPKM values of *RsNAC* genes ^a^Reads Per Kilobase of transcript per Million mapped reads
^b^Days after sowing

10.7717/peerj.4172/supp-10 Table S6 The fold change values of *RsNAC* genes under abiotic stresses Fold change values obtained from transcript levels of 21 randomly selected *RsNAC* genes using real-time polymerase chain reaction.
^a^Polyethylene glycol.
^b^abscisic acid.

## Additional Information and Declarations

### Data Availability

The following information was supplied regarding data availability: The raw data has been supplied as Supplemental Information in Tables S1–S6.
