# Supplementary figures and images for: Genome-wide characterization and expression profiling of NAC transcription factor genes under abiotic stresses in radish (Raphanus sativus L.)
Source: PeerJ. 2017 Dec 15;5:e4172. doi: 10.7717/peerj.4172 (PMC5733918; doi:10.7717/peerj.4172)

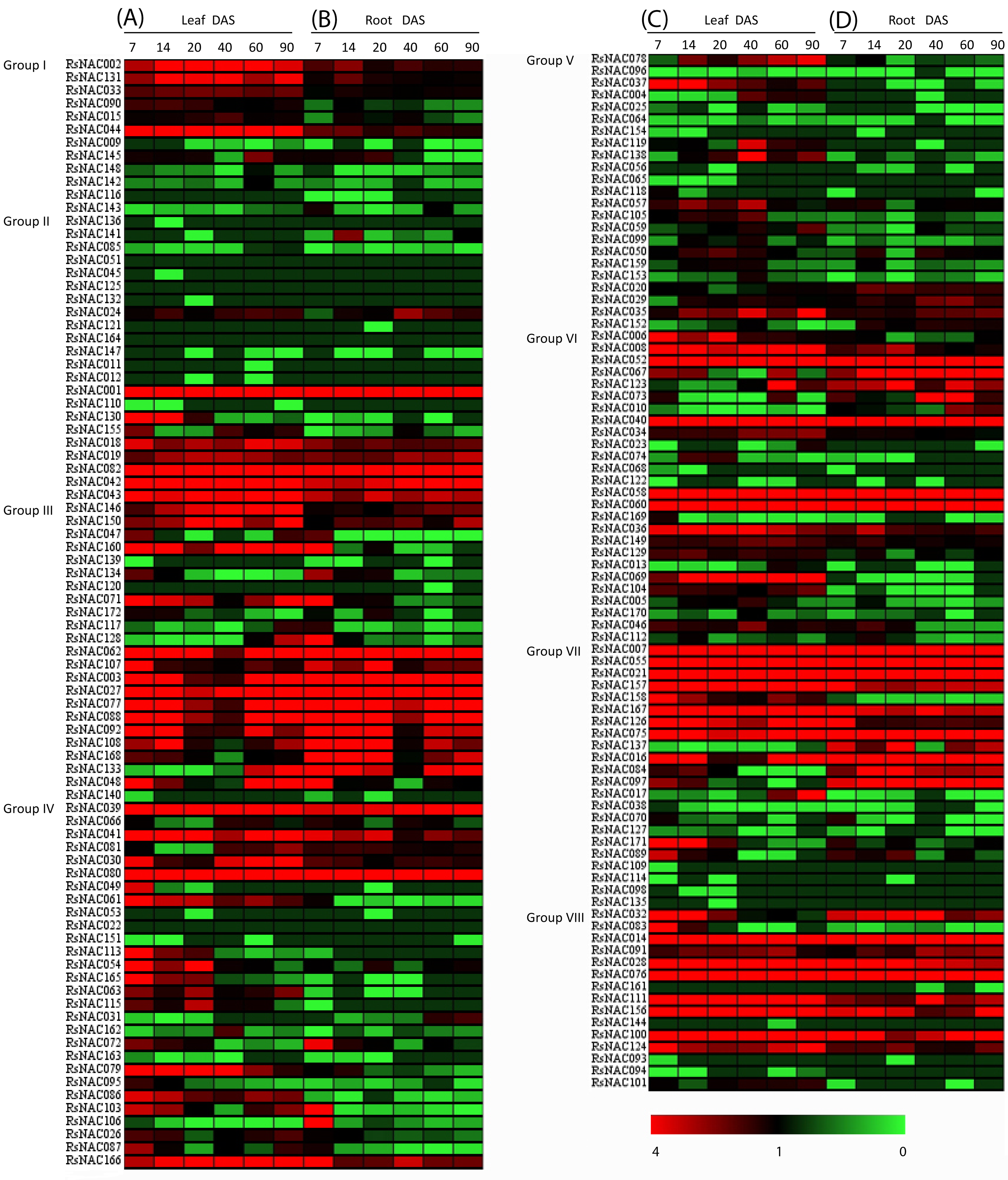

Supplement: Figure S2 — The color scales for fold-change values are shown at the bottom such as elevated (red) and suppressed (green) genes. Several genes were not expressed (black) in any tissue. [file peerj-05-4172-s004.png]
