# Supplementary material for: Genome-wide characterization and expression profiling of NAC transcription factor genes under abiotic stresses in radish (Raphanus sativus L.)
Source: PeerJ. 2017 Dec 15;5:e4172. doi: 10.7717/peerj.4172 (PMC5733918; doi:10.7717/peerj.4172)
Supplement: Table S1 — Gene-specific primers generate from the non-conserved region of NAC sequences using Beacon Designer 7.7. [file peerj-05-4172-s005.docx]

| **Proposed name** | **Forward primer (5’-3’)** | **Reverse primer (5’-3’)** |
| --- | --- | --- |
| *RsNAC145* | GGAGAGTATAATATGAGGAGAG | CAACACCACCTACTATATCA |
| *RsNAC096* | GAGCAAGAAGAAGAGAAGA | AAGACACAAGGACATTAGG |
| *RsNAC041* | TTCCTGTTGAGAGTAACTAC | CCATCTTCCTGAGTATTGT |
| *RsNAC038* | GAGGAATGTGTCTGGTAA | GTTGGTTCTACTCTCATTG |
| *RsNAC040* | TCCTTATGAGTTACCAGAGA | CCTTCTTGTCCTTATCTTGA |
| *RsNAC096* | ATGATGATGATGATGCTGTA | CTGAACACTGACGGTATT |
| *RsNAC082* | AAGACGCAGGAGAATAAC | TGTATTGGAGGAGAGGTAA |
| *RsNAC124* | GGAAGAACCAAGATAGCA | TTACAGCACAAGTCAGAG |
| *RsNAC156* | ATTAGCGAGGTCAACATC | CAATATCCAGAAGGTGTAGT |
| *RsNAC103* | ACGAATGGGTTATTAGTAGAG | TTGAGGTATGTAGAAGAATCC |
| *RsNAC140* | CTAATGACGAGTGTAAGAGA | TGACTATGATGAGGAGGT |
| *RsNAC168* | TTTCCTTCGTTTGATGAC | AGGTCATCTTGCTATACTT |
| *RsNAC041* | TTCCTGTTGAGAGTAACTAC | CCATCTTCCTGAGTATTGT |
| *RsNAC039* | TCGTCTTACTGATGAGGA | CTGCTCTCCATTCTTAGG |
| *RsNAC145* | GGAGAGTATAATATGAGGAGAG | CAACACCACCTACTATATCA |
| *RsNAC058* | CTCCGTTACACTCTCAAG | AGTCTGAATCTGGAAGTTG |
| *RsNAC023* | AAGTCCGTTGAGATTATCC | GTTGCTCTTCTGTTCTGT |
| *RsNAC034* | GTGGTGACATAGCAGAAT | GAATCTCGGCATAGCAAT |
| *RsNAC096* | GAGCAAGAAGAAGAGAAGA | AAGACACAAGGACATTAGG |
| *RsNAC126* | ATGATGATGATGATGCTGTA | CTGAACACTGACGGTATT |
| *RsNAC014* | GGAGAATCAAGCAATAACG | CAGAGTCAACAACACAGT |
| *Actin* | GCATCACACTTTCTACAAC | CCTGGATAGCAACATACAT |
