# Supplementary material for: Genome-wide characterization and expression profiling of NAC transcription factor genes under abiotic stresses in radish (Raphanus sativus L.)
Source: PeerJ. 2017 Dec 15;5:e4172. doi: 10.7717/peerj.4172 (PMC5733918; doi:10.7717/peerj.4172)
Supplement: Table S2 — aTransmembrane motif predicted using the TMHMM server aAmino acid cMolecular weight dIsoelectric points e Arabidopsis thaliana [file peerj-05-4172-s006.docx]

**Table S2.** Characteristic features of NAC transcription factor gene family identified in *R.sativus*

| **Sub group** | **Gene ID** | **Gene Loci** | **^a^TM** | **^b^aa** | **^c^Mw** | **^d^pI** | **^e^At ortholog locus** | **At locus description** |
| --- | --- | --- | --- | --- | --- | --- | --- | --- |
| **Group I** | *RsNAC002* | RSG00243.t1 |  | 306 | 33931.12 | 9.18 | AT4G28500.1 | ANAC073 |
|  | *RsNAC131* | RSG37246.t1 |  | 446 | 51276.91 | 5.07 | AT4G28500.1 | ANAC073 |
|  | *RsNAC033* | RSG10440.t1 |  | 499 | 55129.02 | 6.63 | AT4G28500.1 | ANAC073 |
|  | *RsNAC090* | RSG24224.t1 | 280- 341 | 339 | 36781.52 | 8.71 | AT4G28530.1 | ANAC074 |
|  | *RsNAC015* | RSG02922.t1 |  | 396 | 44523.84 | 6.87 | AT4G29230.1 | ANAC075 |
|  | *RsNAC044* | RSG12009.t1 |  | 314 | 35439.61 | 7.22 | AT5G56620.1 | ANAC099 |
|  | *RsNAC009* | RSG01837.t1 |  | 276 | 31410.52 | 6.66 | AT1G28470.1 | ANAC010 |
|  | *RsNAC145* | RSG43886.t1 | 410- 430 | 428 | 49156.57 | 5.52 | AT5G14490.1 | ANAC085 |
|  | *RsNAC148* | RSG44422.t1 |  | 260 | 29161.39 | 5.79 | AT1G01010.1 | ANAC001 |
|  | *RsNAC142* | RSG43883.t1 |  | 380 | 43036.32 | 5.97 | AT1G02230.1 | ANAC004 |
|  | *RsNAC116* | RSG33043.t1 |  | 399 | 45016.16 | 5.88 | AT1G02230.1 | ANAC004 |
|  | *RsNAC143* | RSG43884.t1 |  | 143 | 16912.09 | 9.27 | AT1G02230.1 | ANAC004 |
| **Group II** | *RsNAC136* | RSG38956.t1 |  | 545 | 62302.15 | 4.86 | AT1G02250.1 | ANAC005 |
|  | *RsNAC141* | RSG42596.t1 |  | 363 | 41268.74 | 5.99 | AT1G03490.1 | ANAC001 |
|  | *RsNAC085* | RSG22905.t1 |  | 552 | 62301.92 | 4.98 | AT1G03490.1 | ANAC001 |
|  | *RsNAC051* | RSG12723.t1 |  | 192 | 22460.45 | 6.91 | AT5G18300.1 | ANAC088 |
|  | *RsNAC045* | RSG12026.t1 |  | 202 | 23299.42 | 6.91 | AT2G02450.1 | ANAC035 |
|  | *RsNAC125* | RSG35639.t1 |  | 202 | 23299.42 | 6.91 | AT2G02450.1 | ANAC035 |
|  | *RsNAC132* | RSG38041.t1 |  | 446 | 51276.91 | 5.07 | AT2G02450.1 | ANAC035 |
|  | *RsNAC024* | RSG08647.t1 |  | 198 | 22921.82 | 9.42 | AT2G02450.1 | ANAC035 |
|  | *RsNAC121* | RSG34803.t1 |  | 538 | 62539.88 | 6.08 | AT3G56530.1 | ANAC064 |
|  | *RsNAC164* | RSG51182.t1 |  | 259 | 30074.83 | 5.64 | AT3G56530.1 | ANAC064 |
|  | *RsNAC147* | RSG44211.t1 |  | 328 | 37560.73 | 4.64 | AT3G56530.1 | ANAC064 |
|  | *RsNAC011* | RSG02257.t1 |  | 532 | 62069.78 | 4.53 | AT1G03490.1 | ANAC001 |
|  | *RsNAC012* | RSG02290.t1 |  | 448 | 51672.55 | 4.69 | AT1G03490.1 | ANAC001 |
|  | *RsNAC001* | RSG00240.t1 |  | 325 | 37172.38 | 5.8 | AT1G03490.1 | ANAC001 |
|  | *RsNAC110* | RSG31173.t1 |  | 348 | 39957.72 | 5.96 | AT4G28530.1 | ANAC074 |
|  | *RsNAC130* | RSG37245.t1 |  | 349 | 40076.81 | 5.7 | AT4G28530.1 | ANAC074 |
|  | *RsNAC155* | RSG48279.t1 |  | 198 | 22981.73 | 4.66 | AT4G28530.1 | ANAC074 |
|  | *RsNAC018* | RSG03795.t1 |  | 459 | 51461.83 | 5.25 | AT5G41090.1 | ANAC095 |
|  | *RsNAC019* | RSG03796.t1 |  | 461 | 51826.36 | 6.41 | AT3G10480.1 | ANAC050 |
|  | *RsNAC082* | RSG22507.t1 |  | 485 | 54562.25 | 5.62 | AT3G10480.1 | ANAC050 |
|  | *RsNAC042* | RSG11916.t1 |  | 443 | 49809.63 | 5.45 | AT3G10480.1 | ANAC050 |
|  | *RsNAC043* | RSG11917.t1 |  | 425 | 47743.335.32 | 5.32 | AT3G10480.1 | ANAC050 |
| **Group III** | *RsNAC146* | RSG43952.t1 | 517- 548 | 546 | 60503.99 | 4.49 | AT3G10480.1 | ANAC050 |
|  | *RsNAC150* | RSG45003.t1 |  | 184 | 21802.23 | 4.71 | AT1G34190.1 | ANAC017 |
|  | *RsNAC047* | RSG12243.t1 |  | 357 | 39243.23 | 8.21 | AT5G50820.1 | ANAC097 |
|  | *RsNAC160* | RSG49514.t1 |  | 362 | 39684.69 | 7.64 | AT3G15510.1 | ANAC056 |
|  |  |  |  |  |  |  |  |  |
| **Table S2**. Continued | | | | | | | | |
|  | *RsNAC139* | RSG39535.t1 |  | 351 | 38723.64 | 8.69 | AT3G15510.1 | ANAC056 |
|  | *RsNAC134* | RSG38365.t1 |  | 237 | 27168.45 | 8.7 | AT3G15510.1 | ANAC056 |
|  | *RsNAC120* | RSG34748.t1 |  | 319 | 35775.06 | 8.8 | AT3G15510.1 | ANAC056 |
|  | *RsNAC071* | RSG19371.t1 |  | 356 | 40776.79 | 7.2 | AT1G61110.1 | ANAC025 |
|  | *RsNAC172* | RSG56445.t1 |  | 174 | 19804.58 | 9.63 | AT3G04070.1 | ANAC047 |
|  | *RsNAC117* | RSG33856.t1 |  | 271 | 1449.72 | 6.4 | AT3G04070.1 | ANAC047 |
|  | *RsNAC128* | RSG36727.t1 |  | 271 | 31449.72 | 6.4 | AT1G69490.1 | ANAC029 |
|  | *RsNAC062* | RSG16872.t1 |  | 292 | 32937.37 | 6.16 | AT1G69490.1 | ANAC029 |
|  | *RsNAC107* | RSG30206.t1 |  | 261 | 29935.25 | 9.1 | AT1G01720.1 | ANAC002 |
|  | *RsNAC003* | RSG00723.t1 |  | 410 | 45979.37 | 5.68 | AT1G77450.1 | ANAC032 |
|  | *RsNAC027* | RSG08857.t1 |  | 197 | 22693.87 | 9.33 | AT5G08790.1 | ANAC081 |
|  | *RsNAC077* | RSG20033.t1 |  | 280 | 31773.67 | 6.14 | AT5G08790.1 | ANAC081 |
|  | *RsNAC088* | RSG23615.t1 |  | 266 | 30287.47 | 8.14 | AT5G08790.1 | ANAC081 |
|  | *RsNAC092* | RSG25363.t1 |  | 253 | 28886.5 | 6.66 | AT5G63790.1 | ANAC102 |
|  | *RsNAC108* | RSG30854.t1 |  | 300 | 33237.99 | 8.37 | AT5G63790.1 | ANAC102 |
|  | *RsNAC168* | RSG52993.t1 |  | 271 | 30451.36 | 8.54 | AT1G52890.1 | ANAC019 |
|  | *RsNAC133* | RSG38363.t1 |  | 314 | 35235.1 | 5.45 | AT1G52890.1 | ANAC019 |
|  | *RsNAC048* | RSG12244.t1 |  | 333 | 37416.67 | 8.32 | AT1G52890.1 | ANAC019 |
|  | *RsNAC140* | RSG39697.t1 |  | 317 | 35859.78 | 6.32 | AT3G15500.1 | ANAC055 |
| **Group IV** | *RsNAC039* | RSG11497.t1 | 339- 370 | 568 | 63644.44 | 4.55 | AT3G15500.1 | ANAC055 |
|  | *RsNAC066* | RSG17948.t1 |  | 185 | 21353.7 | 4.91 | AT5G04410.1 | NAC002 |
|  | *RsNAC041* | RSG11915.t1 | 509- 537 | 535 | 60203.18 | 4.56 | AT5G64530.1 | ANAC104 |
|  | *RsNAC081* | RSG22217.t1 |  | 188 | 21540.89 | 4.83 | AT3G10500.1 | ANAC053 |
|  | *RsNAC030* | RSG09412.t1 |  | 570 | 63387.93 | 4.44 | AT5G64530.1 | ANAC104 |
|  | *RsNAC080* | RSG22125.t1 | 544- 572 | 188 | 21550.83 | 4.77 | AT5G64530.1 | ANAC104 |
|  | *RsNAC049* | RSG12277.t1 |  | 314 | 35332.15 | 7.63 | AT5G04410.1 | NAC2 |
|  | *RsNAC061* | RSG16488.t1 |  | 311 | 34528.05 | 8.16 | AT3G15170.1 | ANAC054 |
|  | *RsNAC053* | RSG13541.t1 |  | 314 | 34980.61 | 6.96 | AT3G15170.1 | ANAC054 |
|  | *RsNAC022* | RSG05699.t1 |  | 367 | 40772.49 | 8.82 | AT3G15170.1 | ANAC054 |
|  | *RsNAC151* | RSG45507.t1 |  | 368 | 40661.54 | 8.75 | AT5G53950.1 | ANAC098 |
|  | *RsNAC113* | RSG31961.t1 |  | 149 | 17382.79 | 8.98 | AT5G53950.1 | ANAC098 |
|  | *RsNAC054* | RSG13739.t1 |  | 299 | 33948.11 | 6.54 | AT3G18400.1 | ANAC058 |
|  | *RsNAC165* | RSG51320.t1 |  | 308 | 34986.28 | 6.02 | AT3G18400.1 | ANAC058 |
|  | *RsNAC063* | RSG17079.t1 |  | 316 | 35520.82 | 8.64 | AT3G18400.1 | ANAC058 |
|  | *RsNAC115* | RSG32951.t1 |  | 317 | 35914.09 | 7.81 | AT2G24430.1 | ANAC038 |
|  | *RsNAC031* | RSG09835.t1 |  | 335 | 37980.62 | 6.04 | AT2G24430.1 | ANAC038 |
|  | *RsNAC162* | RSG51048.t1 |  | 320 | 36363.99 | 6.06 | AT1G76420.1 | ANAC031 |
|  | *RsNAC072* | RSG19372.t1 |  | 342 | 38002.5 | 5.87 | AT1G76420.1 | ANAC031 |
|  | *RsNAC163* | RSG51109.t1 |  | 326 | 36584 | 5.81 | AT3G04060.1 | ANAC046 |
|  |  |  |  |  |  |  |  |  |
| **Table S2**. Continued | | | | | | | | |
|  | *RsNAC079* | RSG21630.t1 |  | 337 | 38490.53 | 5.96 | AT3G04060.1 | ANAC046 |
|  | *RsNAC095* | RSG26111.t1 |  | 316 | 36200.61 | 6.36 | AT5G18270.1 | ANAC087 |
|  | *RsNAC086* | RSG23023.t1 |  | 300 | 34567.27 | 6.14 | AT5G18270.1 | ANAC087 |
|  | *RsNAC103* | RSG28433.t1 |  | 326 | 37046.05 | 6.77 | AT5G39610.1 | ANAC092 |
|  | *RsNAC106* | RSG29844.t1 |  | 309 | 34786.73 | 6.55 | AT3G29035.1 | ANAC059 |
|  | *RsNAC026* | RSG08804.t1 |  | 238 | 27163.99 | 9.34 | AT3G29035.1 | ANAC059 |
|  | *RsNAC087* | RSG23385.t1 |  | 243 | 27943.11 | 9.15 | AT5G07680.1 | ANAC079 |
|  | *RsNAC166* | RSG52719.t1 |  | 324 | 37154.08 | 8.61 | AT5G61430.1 | ANAC100 |
|  | *RsNAC078* | RSG20769.t1 |  | 325 | 37425.49 | 5.9 | AT5G61430.1 | ANAC100 |
| **Group V** | *RsNAC096* | RSG27552.t1 | 309-334 and 346-424 | 444 | 50566.78 | 7.18 | AT1G71930.1 | ANAC030 |
|  | *RsNAC037* | RSG11079.t1 |  | 275 | 31701.7 | 8.96 | AT1G71930.1 | ANAC030 |
|  | *RsNAC004* | RSG01200.t1 |  | 359 | 40310.04 | 5.72 | AT3G12977.1 | AT3G12977.1 |
|  | *RsNAC025* | RSG08749.t1 |  | 367 | 41252.06 | 5.56 | AT2G46770.1 | ANAC070 |
|  | *RsNAC064* | RSG17257.t1 |  | 325 | 37243.92 | 5.57 | AT2G46770.1 | ANAC043 |
|  | *RsNAC154* | RSG46630.t1 |  | 333 | 38024.84 | 6.26 | AT3G61910.1 | ANAC066 |
|  | *RsNAC119* | RSG34595.t1 |  | 360 | 41526.37 | 5.41 | AT3G61910.1 | ANAC066 |
|  | *RsNAC138* | RSG39304.t1 |  | 365 | 42611.46 | 5.94 | AT1G32770.1 | ANAC012 |
|  | *RsNAC056* | RSG14554.t1 |  | 341 | 39150.53 | 6.6 | AT1G32770.1 | ANAC012 |
|  | *RsNAC065* | RSG17700.t1 |  | 356 | 41024.18 | 6.76 | AT4G10350.1 | ANAC070 |
|  | *RsNAC118* | RSG33885.t1 |  | 361 | 41474.77 | 6.63 | AT1G79580.1 | ANAC033 |
|  | *RsNAC057* | RSG14848.t1 |  | 392 | 45315.37 | 5.83 | AT1G79580.1 | ANAC033 |
|  | *RsNAC105* | RSG28764.t1 |  | 391 | 45530.25 | 5.61 | AT1G12260.1 | ANAC007 |
|  | *RsNAC059* | RSG15654.t1 |  | 398 | 46456.32 | 6.47 | AT1G12260.1 | ANAC007 |
|  | *RsNAC099* | RSG28351.t1 |  | 396 | 45892.59 | 5.69 | AT1G62700.1 | ANAC026 |
|  | *RsNAC050* | RSG12456.t1 |  | 348 | 39710.04 | 5.08 | AT1G12260.1 | ANAC007 |
|  | *RsNAC159* | RSG49510.t1 |  | 350 | 40172.35 | 5.19 | AT5G62380.1 | ANAC101 |
|  | *RsNAC153* | RSG46518.t1 |  | 317 | 36313.2 | 5.08 | AT5G62380.1 | ANAC101 |
|  | *RsNAC020* | RSG04122.t1 |  | 377 | 43664.9 | 5.8 | AT5G62380.1 | ANAC101 |
|  | *RsNAC029* | RSG08956.t1 |  | 370 | 42963.22 | 6.16 | AT4G36160.1 | ANAC076 |
|  | *RsNAC035* | RSG10550.t1 |  | 295 | 34071.51 | 5.7 | AT4G36160.1 | ANAC076 |
|  | *RsNAC152* | RSG45857.t1 |  | 361 | 42162.43 | 5.81 | AT5G66300.1 | ANAC105 |
| **Group VI** | *RsNAC006* | RSG01548.t1 |  | 212 | 24054.28 | 9.27 | AT2G18060.1 | ANAC037 |
|  | *RsNAC008* | RSG01805.t1 |  | 206 | 23487.71 | 9.47 | AT5G14000.1 | ANAC084 |
|  | *RsNAC052* | RSG13466.t1 |  | 471 | 52093.68 | 5.4 | AT5G14000.1 | ANAC084 |
|  | *RsNAC067* | RSG18153.t1 |  | 234 | 26466.85 | 5.67 | AT3G49530.1 | ANAC062 |
|  | *RsNAC123* | RSG35036.t1 |  | 150 | 17106.49 | 9.04 | AT5G22380.1 | ANAC090 |
|  | *RsNAC073* | RSG19414.t1 |  | 235 | 26845.45 | 6.93 | AT5G22380.1 | ANAC090 |
|  | *RsNAC010* | RSG02101.t1 |  | 280 | 31322.3 | 8.7 | AT5G22380.1 | ANAC090 |
|  | *RsNAC040* | RSG11762.t1 |  | 458 | 51289.01 | 5.71 | AT3G44350.1 | ANAC061 |
|  |  |  |  |  |  |  |  |  |
| **Table S2**. Continued | | | | | | | | |
|  | *RsNAC034* | RSG10501.t1 | 322-351 | 349 | 39626.49 | 5.41 | AT3G49530.1 | ANAC062 |
|  | *RsNAC023* | RSG07643.t1 | 316-337 | 335 | 37766.02 | 5.67 | AT2G27300.1 | ANAC040 |
|  | *RsNAC074* | RSG19420.t1 |  | 456 | 51715.95 | 5.83 | AT3G44290.1 | ANAC060 |
|  | *RsNAC068* | RSG18158.t1 |  | 335 | 37985.25 | 5.12 | AT5G22290.1 | ANAC089 |
|  | *RsNAC122* | RSG35030.t1 |  | 347 | 38795.99 | 5.15 | AT5G22290.1 | ANAC089 |
|  | *RsNAC058* | RSG14996.t1 | 408-442 | 440 | 49403.73 | 8.27 | AT5G22290.1 | ANAC089 |
|  | *RsNAC060* | RSG16412.t1 |  | 453 | 50425.68 | 6.43 | AT3G49530.1 | ANAC062 |
|  | *RsNAC169* | RSG53660.t1 |  | 282 | 32550.34 | 5.49 | AT3G49530.1 | ANAC062 |
|  | *RsNAC036* | RSG10766.t1 |  | 244 | 27961.08 | 4.89 | AT1G32510.1 | ANAC011 |
|  | *RsNAC149* | RSG44690.t1 |  | 547 | 62020.42 | 6.08 | AT3G17730.1 | ANAC057 |
|  | *RsNAC129* | RSG36770.t1 |  | 309 | 35772.27 | 5.27 | AT1G65910.1 | ANAC028 |
|  | *RsNAC013* | RSG02614.t1 |  | 286 | 32778.85 | 6.95 | AT1G54330.1 | ANAC020 |
|  | *RsNAC069* | RSG18827.t1 |  | 289 | 33208.2 | 8.1 | AT4G17980.1 | ANAC071 |
|  | *RsNAC104* | RSG28487.t1 |  | 296 | 34093.12 | 6.19 | AT4G17980.1 | ANAC071 |
|  | *RsNAC005* | RSG01327.t1 | 2968 | 498 | 57140.83 | 5.45 | AT5G46590.1 | ANAC096 |
|  | *RsNAC170* | RSG53820.t1 |  | 153 | 18011.66 | 9.59 | AT5G17260.1 | ANAC086 |
|  | *RsNAC046* | RSG12094.t1 |  | 478 | 55083.31 | 5.41 | AT5G17260.1 | ANAC086 |
|  | *RsNAC112* | RSG31855.t1 |  | 390 | 44970.46 | 5.16 | AT3G03200.1 | ANAC045 |
| **Group VII** | *RsNAC007* | RSG01763.t1 |  | 249 | 28309.88 | 9.11 | AT3G03200.1 | ANAC045 |
|  | *RsNAC055* | RSG14482.t1 |  | 248 | 28129.54 | 9.05 | AT5G13180.1 | ANAC083 |
|  | *RsNAC021* | RSG05613.t1 |  | 249 | 28421.05 | 9.2 | AT5G13180.1 | ANAC083 |
|  | *RsNAC157* | RSG48570.t1 |  | 429 | 48687.09 | 4.45 | AT5G13180.1 | ANAC083 |
|  | *RsNAC158* | RSG48659.t1 | 541-575 | 573 | 64345.88 | 5.08 | AT1G34190.1 | ANAC017 |
|  | *RsNAC167* | RSG52916.t1 |  | 350 | 39877.52 | 4.67 | AT1G34180.1 | ANAC016 |
|  | *RsNAC126* | RSG35848.t1 | 534-567 | 565 | 63008.46 | 5.29 | AT1G34190.1 | ANAC017 |
|  | *RsNAC075* | RSG19838.t1 |  | 285 | 32917.68 | 8.71 | AT1G34180.1 | ANAC016 |
|  | *RsNAC137* | RSG38993.t1 |  | 285 | 33113.15 | 9.18 | AT2G17040.1 | ANAC036 |
|  | *RsNAC016* | RSG03030.t1 |  | 276 | 31833.52 | 7.66 | AT2G17040.1 | ANAC036 |
|  | *RsNAC084* | RSG22781.t1 |  | 397 | 45515.03 | 5.88 | AT2G17040.1 | ANAC036 |
|  | *RsNAC097* | RSG27764.t1 |  | 416 | 47213.9 | 6.05 | AT2G02450.1 | ANAC034 |
|  | *RsNAC017* | RSG03678.t1 |  | 304 | 35043.35 | 8.95 | AT2G02450.1 | ANAC035 |
|  | *RsNAC038* | RSG11087.t1 |  | 311 | 35975.55 | 8.88 | AT3G12910.1 | AT3G12910.1 |
|  | *RsNAC070* | RSG18911.t1 |  | 271 | 31193.01 | 0.67 | AT3G12910.1 | ANAC042 |
|  | *RsNAC127* | RSG35960.t1 |  | 245 | 27995.04 | 5.66 | AT2G43000.1 | ANAC042 |
|  | *RsNAC171* | RSG55051.t1 |  | 154 | 17938.45 | 9.11 | AT2G43000.1 | ANAC042 |
|  | *RsNAC089* | RSG24155.t1 |  | 277 | 31913.9 | 8.75 | AT2G43000.1 | ANAC094 |
|  | *RsNAC109* | RSG31027.t1 |  | 335 | 38090.49 | 8.26 | AT5G39820.1 | ANAC009 |
|  | *RsNAC114* | RSG32015.t1 |  | 378 | 42757.98 | 7.66 | AT1G26870.1 | ANAC009 |
|  | *RsNAC098* | RSG27884.t1 |  | 153 | 17949.46 | 9.39 | AT1G26870.1 | ANAC009 |
|  |  |  |  |  |  |  |  |  |
| **Table S2**. Continued | | | | | | | | |
|  | RsNAC135 | RSG38941.t1 |  | 417 | 47356.03 | 6.06 | AT1G26870.1 | ANAC041 |
| **Group VIII** | RsNAC032 | RSG10059.t1 |  | 256 | 29266.23 | 8.73 | AT2G33480.1 | ANAC041 |
|  | RsNAC083 | RSG22714.t1 |  | 254 | 29374 | 9.07 | AT2G33480.1 | ANAC078 |
|  | RsNAC014 | RSG02694.t1 | 521-549 | 547 | 60744.2 | 4.52 | AT5G04410.1 | ANAC078 |
|  | RsNAC091 | RSG25336.t1 |  | 345 | 38748.6 | 4.96 | AT5G64060.1 | ANAC103 |
|  | RsNAC028 | RSG08863.t1 |  | 399 | 44317.33 | 4.58 | AT5G09330.1 | ANAC082 |
|  | RsNAC076 | RSG20023.t1 |  | 427 | 47184.47 | 4.9 | AT5G09330.1 | ANAC082 |
|  | RsNAC161 | RSG49516.t1 |  | 210 | 23747.13 | 5.84 | AT5G64060.1 | ANAC103 |
|  | RsNAC111 | RSG31666.t1 | 611-654 | 652 | 73746.83 | 5.75 | AT1G33060.1 | ANAC014 |
|  | RsNAC156 | RSG48380.t1 |  | 149 | 17461.16 | 9.45 | AT4G01550.1 | ANAC069 |
|  | RsNAC144 | RSG43885.t1 |  | 296 | 33786.72 | 6.08 | AT3G04430.1 | ANAC049 |
|  | RsNAC100 | RSG28379.t1 |  | 146 | 16890.3 | 9.38 | AT4G01550.1 | ANAC069 |
|  | RsNAC124 | RSG35391.t1 | 480-511 | 509 | 56626.75 | 5.19 | AT1G32870.1 | ANAC013 |
|  | RsNAC093 | RSG25670.t1 |  | 291 | 32914.31 | 5.91 | AT4G01550.1 | ANAC069 |
|  | RsNAC094 | RSG25672.t1 |  | 287 | 32573.85 | 5.23 | AT4G01550.1 | ANAC069 |
|  | RsNAC101 | RSG28382.t1 |  | 171 | 19601.72 | 9.13 | AT4G01550.1 | ANAC069 |
|  | RsNAC102 | RSG28385.t1 |  | 360 | 38748.6 | 9.11 | AT4G01550.1 | ANAC069 |
