# Supplementary material for: Genome-wide characterization and expression profiling of NAC transcription factor genes under abiotic stresses in radish (Raphanus sativus L.)
Source: PeerJ. 2017 Dec 15;5:e4172. doi: 10.7717/peerj.4172 (PMC5733918; doi:10.7717/peerj.4172)
Supplement: Table S4 — *Megabase [file peerj-05-4172-s008.docx]

**Table S4.** The information of linkage group localization of *NAC* genes.

| **Gene ID** | **Position (Mb*)** | **Chromosome** | **Total length** |
| --- | --- | --- | --- |
| *RsNAC044* | 12.68 | R1 | 26.31 |
| *RsNAC116* | 0.70 |  |  |
| *RsNAC143* | 0.69 |  |  |
| *RsNAC134* | 24.96 |  |  |
| *RsNAC133* | 24.98 |  |  |
| *RsNAC138* | 15.41 |  |  |
| *RsNAC129* | 26.28 |  |  |
| *RsNAC135* | 11.11 |  |  |
| *RsNAC167* | 16.66 |  |  |
| *RsNAC002* | 38.67 | R2 | 43.80 |
| *RsNAC131* | 3.72 |  |  |
| *RsNAC162* | 1.93 |  |  |
| *RsNAC026* | 28.22 |  |  |
| *RsNAC122* | 21.60 |  |  |
| *RsNAC149* | 8.95 |  |  |
| *RsNAC055* | 29.74 |  |  |
| *RsNAC028* | 28.59 |  |  |
| *RsNAC156* | 13.79 |  |  |
| *RsNAC107* | 1.56 |  |  |
| *RsNAC027* | 28.57 |  |  |
| *RsNAC067* | 23.61 | R3 | 29.13 |
| *RsNAC007* | 26.16 |  |  |
| *RsNAC068* | 23.63 |  |  |
| *RsNAC130* | 3.70 | R4 | 50.00 |
| *RsNAC108* | 4.11 |  |  |
| *RsNAC115* | 32.63 |  |  |
| *RsNAC087* | 9.93 |  |  |
| *RsNAC025* | 43.09 |  |  |
| *RsNAC029* | 1.14 |  |  |
| *RsNAC097* | 17.09 |  |  |
| *RsNAC089* | 41.44 |  |  |
| *RsNAC032* | 37.16 |  |  |
| *RsNAC024* | 29.35 | R5 | 45.94 |
| *RsNAC019* | 43.89 |  |  |
| *RsNAC146* | 22.77 |  |  |
| *RsNAC047* | 7.24 |  |  |
| *RsNAC139* | 0.02 |  |  |
| *RsNAC172* | 1.21 |  |  |
| *RsNAC048* | 7.22 |  |  |
| *RsNAC081* | 39.14 |  |  |
| *RsNAC049* | 6.99 |  |  |
| *RsNAC163* | 1.21 |  |  |
| *RsNAC119* | 22.42 |  |  |
| *RsNAC057* | 35.36 |  |  |
|  |  |  |  |
| **Table S4. Continued** | | | |
| *RsNAC050* | 36.60 |  |  |
| *RsNAC010* | 27.34 |  |  |
| *RsNAC036* | 8.45 |  |  |
| *RsNAC158* | 22.76 |  |  |
| *RsNAC017* | 43.20 |  |  |
| *RsNAC038* | 5.54 |  |  |
| *RsNAC161* | 11.99 |  |  |
| *RsNAC111* | 22.51 |  |  |
| *RsNAC112* | 0.80 |  |  |
| *RsNAC145* | 20.52 | R6 | 53.64 |
| *RsNAC160* | 5.77 |  |  |
| *RsNAC071* | 1.02 |  |  |
| *RsNAC088* | 12.91 |  |  |
| *RsNAC140* | 5.72 |  |  |
| *RsNAC066* | 12.63 |  |  |
| *RsNAC041* | 3.01 |  |  |
| *RsNAC053* | 4.97 |  |  |
| *RsNAC113* | 7.07 |  |  |
| *RsNAC063* | 39.80 |  |  |
| *RsNAC072* | 1.01 |  |  |
| *RsNAC166* | 13.42 |  |  |
| *RsNAC159* | 13.14 |  |  |
| *RsNAC152* | 25.53 |  |  |
| *RsNAC034* | 43.89 |  |  |
| *RsNAC060* | 37.25 |  |  |
| *RsNAC104* | 21.69 |  |  |
| *RsNAC137* | 26.79 |  |  |
| *RsNAC171* | 51.37 |  |  |
| *RsNAC124* | 30.31 |  |  |
| *RsNAC147* | 20.45 | R7 | 27.19 |
| *RsNAC128* | 13.16 |  |  |
| *RsNAC077* | 7.02 |  |  |
| *RsNAC039* | 2.54 |  |  |
| *RsNAC031* | 9.09 |  |  |
| *RsNAC006* | 4.94 |  |  |
| *RsNAC074* | 1.89 |  |  |
| *RsNAC005* | 3.72 |  |  |
| *RsNAC170* | 3.72 |  |  |
| *RsNAC021* | 5.47 |  |  |
| *RsNAC076* | 6.98 |  |  |
| *RsNAC110* | 11.34 | R8 | 29.68 |
| *RsNAC022* | 22.40 |  |  |
| *RsNAC151* | 22.40 |  |  |
| *RsNAC169* | 20.46 |  |  |
| *RsNAC157* | 21.01 |  |  |
| *RsNAC098* | 5.64 |  |  |
|  |  |  |  |
| **Table S4. Continued** | | | |
| *RsNAC150* | 0.02 | R9 | 38.35 |
| *RsNAC117* | 2.29 |  |  |
| *RsNAC103* | 31.77 |  |  |
| *RsNAC078* | 4.65 |  |  |
| *RsNAC059* | 28.79 |  |  |
| *RsNAC040* | 30.55 |  |  |
| *RsNAC030* | 37.28 |  |  |
| *RsNAC114* | 2.12 |  |  |
