# Supplementary material for: Genome-wide characterization and expression profiling of NAC transcription factor genes under abiotic stresses in radish (Raphanus sativus L.)
Source: PeerJ. 2017 Dec 15;5:e4172. doi: 10.7717/peerj.4172 (PMC5733918; doi:10.7717/peerj.4172)
Supplement: Table S5 — aReads Per Kilobase of transcript per Million mapped reads bDays after sowing [file peerj-05-4172-s009.docx]

| **Table S5.** The ^a^RPKM values of *RsNAC* genes | | | | | | | | | | | | | |
| --- | --- | --- | --- | --- | --- | --- | --- | --- | --- | --- | --- | --- | --- |
|  | | **Roots** | | | | | | **Leaves** | | | | | |
| **Feature ID** | **Gene ID** | **7 DAS^b^** | **14 DAS** | **20 DAS** | **40 DAS** | **60 DAS** | **90 DAS** | **7 DAS** | **14 DAS** | **20 DAS** | **40 DAS** | **60 DAS** | **90 DAS** |
| RSG00243.t1 | *RsNAC002* | 7.951008 | 16.21257 | 14.04084 | 26.12376 | 12.34373 | 9.582957 | 4.116237 | 6.374388 | 2.230731 | 4.023829 | 2.76637 | 2.85584 |
| RSG37246.t1 | *RsNAC131* | 6.760951 | 11.7337 | 10.51069 | 20.29144 | 7.352485 | 10.55874 | 1.861534 | 5.09739 | 2.286377 | 1.845012 | 1.49444 | 1.640425 |
| RSG10440.t1 | *RsNAC033* | 4.350566 | 5.131122 | 5.603659 | 5.490219 | 4.708273 | 5.225413 | 1.221745 | 2.244361 | 1.631111 | 1.408259 | 1.740915 | 1.919116 |
| RSG24224.t1 | *RsNAC090* | 3.534414 | 3.867293 | 3.191208 | 1.236416 | 1.296756 | 2.075206 | 0.684945 | 1.252619 | 0.945882 | 1.027474 | 0.597291 | 0.555766 |
| RSG02922.t1 | *RsNAC015* | 1.636862 | 1.84587 | 2.574315 | 4.052101 | 1.527243 | 1.8391 | 0.553041 | 1.8261 | 1.147158 | 1.656129 | 0.922312 | 0.576406 |
| RSG12009.t1 | *RsNAC044* | 31.48342 | 22.89373 | 26.46568 | 25.21641 | 14.76112 | 11.25155 | 4.895058 | 5.147637 | 2.695317 | 4.365416 | 3.056116 | 2.55383 |
| RSG01837.t1 | *RsNAC009* | 0 | 0 | 0.15803 | 0.267127 | 0.056057 | 0.516454 | 0.085215 | 0 | 0.056058 | 0 | 0.018675 | 0.088464 |
| RSG43886.t1 | *RsNAC145* | 1.992423 | 1.760315 | 2.028053 | 0.390482 | 5.786958 | 1.931673 | 1.862183 | 2.276131 | 3.48524 | 0 | 0.066041 | 0.070489 |
| RSG44422.t1 | *RsNAC148* | 0.78679 | 0.555037 | 0.569717 | 0.09542 | 1.180381 | 0.442738 | 0 | 0.092566 | 0.143877 | 0.178169 | 0.431694 | 0.68323 |
| RSG43883.t1 | *RsNAC142* | 0.596671 | 0.558521 | 0.716852 | 0.341524 | 1.254667 | 0.497077 | 0.603607 | 0.314853 | 0.357921 | 0 | 0.27827 | 0.306664 |
| RSG33043.t1 | *RsNAC116* | 0 | 0 | 0 | 0 | 0 | 0 | 0.030622 | 0.03079 | 0.057181 | 0 | 0 | 0 |
| RSG43884.t1 | *RsNAC143* | 0.164773 | 0.321325 | 0.359832 | 0.217093 | 0.690233 | 0.79468 | 2.072403 | 0.314055 | 0.079418 | 0.322931 | 1.302501 | 0.465255 |
| RSG38956.t1 | *RsNAC136* | 0 | 0.021106 | 0 | 0 | 0 | 0 | 0 | 0 | 0 | 0 | 0 | 0 |
| RSG42596.t1 | *RsNAC141* | 0 | 0 | 0.048632 | 0 | 0 | 0 | 0.382473 | 5.916056 | 0.291649 | 0.696928 | 0.452569 | 1.328782 |
| RSG22905.t1 | *RsNAC085* | 0.415829 | 0.17813 | 0.190655 | 0.202517 | 0.972971 | 1.040354 | 0.042767 | 0.357358 | 0.106197 | 0.214254 | 0.308283 | 0.098995 |
| RSG12723.t1 | *RsNAC051* | 0 | 0 | 0 | 0 | 0 | 0 | 0 | 0 | 0 | 0 | 0 | 0 |
| RSG12026.t1 | *RsNAC045* | 0 | 0.063354 | 0 | 0 | 0 | 0 | 0 | 0 | 0 | 0 | 0 | 0 |
| RSG35639.t1 | *RsNAC125* | 0 | 0 | 0 | 0 | 0 | 0 | 0 | 0 | 0 | 0 | 0 | 0 |
| RSG38041.t1 | *RsNAC132* | 0 | 0 | 0.053564 | 0 | 0 | 0 | 0 | 0 | 0 | 0 | 0 | 0 |
| RSG08647.t1 | *RsNAC024* | 1.640553 | 3.534621 | 1.451183 | 3.206292 | 3.960744 | 3.404001 | 0.7946 | 2.015099 | 1.482569 | 7.128073 | 4.561555 | 2.951229 |
| RSG34803.t1 | *RsNAC121* | 0 | 0 | 0 | 0 | 0 | 0 | 0 | 0 | 0.019643 | 0 | 0 | 0 |
| RSG51182.t1 | *RsNAC164* | 0 | 0 | 0 | 0 | 0 | 0 | 0 | 0 | 0 | 0 | 0 | 0 |
| RSG44211.t1 | *RsNAC147* | 0 | 0 | 0.036387 | 0 | 0.129636 | 0.033824 | 0 | 0.036 | 0.034761 | 0 | 0.08137 | 0.074482 |
| RSG02257.t1 | *RsNAC011* | 0 | 0 | 0 | 0 | 0.038664 | 0 | 0 | 0 | 0 | 0 | 0 | 0 |
| RSG02290.t1 | *RsNAC012* | 0 | 0 | 0.022503 | 0 | 0.05573 | 0 | 0 | 0 | 0 | 0 | 0 | 0 |
| RSG00239.t1 | *RsNAC001* | 38.69882 | 32.06676 | 39.46767 | 35.46857 | 35.75466 | 31.00509 | 20.52843 | 20.8516 | 19.55239 | 26.282 | 20.97521 | 19.31883 |
| RSG31173.t1 | *RsNAC110* | 0.072995 | 0.033271 | 0 | 0 | 0 | 0.031886 | 0 | 0 | 0 | 0 | 0 | 0 |
|  |  |  |  |  |  |  |  |  |  |  |  |  |  |
| **Table S5.** Continued | | | | | | | | | | | | | |
| RSG31173.t1 | *RsNAC110* | 0.072995 | 0.033271 | 0 | 0 | 0 | 0.031886 | 0 | 0 | 0 | 0 | 0 | 0 |
| RSG37245.t1 | *RsNAC130* | 22.15817 | 13.20166 | 3.129705 | 0.40455 | 0.345233 | 0.76868 | 0.034997 | 0.331199 | 0.139966 | 0 | 0.01478 | 0 |
| RSG48279.t1 | *RsNAC155* | 5.676824 | 0.412766 | 0.498449 | 3.93527 | 1.71113 | 2.995815 | 0.057292 | 0.333106 | 0.500874 | 1.549175 | 0.745277 | 0.355134 |
| RSG03795.t1 | *RsNAC018* | 9.799773 | 5.797064 | 7.891708 | 6.308937 | 10.26947 | 9.541714 | 6.3738 | 3.936231 | 5.146683 | 4.028512 | 4.315708 | 5.018856 |
| RSG03796.t1 | *RsNAC019* | 4.483301 | 7.226651 | 8.212842 | 8.024133 | 7.140193 | 5.079233 | 5.70069 | 4.797257 | 4.644371 | 7.208205 | 6.822961 | 8.606306 |
| RSG22507.t1 | *RsNAC082* | 17.6083 | 21.87028 | 25.34079 | 18.68357 | 24.53208 | 25.51801 | 11.91949 | 11.72662 | 13.92129 | 13.82359 | 12.61714 | 12.73546 |
| RSG11916.t1 | *RsNAC042* | 24.51956 | 20.86369 | 30.82412 | 23.79377 | 34.73778 | 41.21141 | 9.183569 | 8.242534 | 9.963253 | 12.31249 | 11.77144 | 12.77027 |
| RSG11917.t1 | *RsNAC043* | 13.94055 | 9.965809 | 10.9469 | 10.46368 | 11.89646 | 11.52582 | 8.352981 | 5.447212 | 9.467164 | 6.908551 | 8.690766 | 7.698847 |
| RSG43952.t1 | *RsNAC146* | 3.754074 | 7.862282 | 10.02434 | 13.1062 | 13.62826 | 17.59424 | 1.767852 | 2.381292 | 1.342538 | 3.63365 | 5.465498 | 5.297767 |
| RSG45003.t1 | *RsNAC150* | 5.804326 | 7.057701 | 37.52196 | 17.81064 | 6.370932 | 19.47041 | 1.276659 | 4.290644 | 3.786464 | 5.204507 | 1.850954 | 7.974017 |
| RSG12243.t1 | *RsNAC047* | 5.401046 | 1.012087 | 0.082887 | 0 | 0.227206 | 3.458237 | 4.520175 | 0.068804 | 0.029574 | 0.058334 | 0.014449 | 0.034224 |
| RSG49514.t1 | *RsNAC160* | 18.81301 | 11.47239 | 5.480185 | 18.05772 | 30.2733 | 51.60494 | 14.05766 | 0.674917 | 1.68627 | 0.224382 | 0.301099 | 0.965155 |
| RSG39535.t1 | *RsNAC139* | 0.036186 | 0 | 0 | 0 | 0 | 0 | 0.131966 | 0.034988 | 0 | 0 | 0.014696 | 0 |
| RSG38365.t1 | *RsNAC134* | 4.700166 | 1.257933 | 0.320252 | 0.018808 | 0.052189 | 0.217148 | 7.108577 | 1.679096 | 2.08647 | 0.274128 | 0.636564 | 0.679363 |
| RSG34748.t1 | *RsNAC120* | 0 | 0 | 0 | 0 | 0 | 0 | 0 | 0 | 0 | 0 | 0.016165 | 0 |
| RSG19371.t1 | *RsNAC071* | 17.05532 | 9.57752 | 7.798944 | 1.465706 | 6.431321 | 10.97343 | 18.79632 | 1.702699 | 1.875988 | 0.535101 | 0.592521 | 0.801672 |
| RSG56445.t1 | *RsNAC172* | 2.036538 | 2.114089 | 0.760255 | 0 | 0.356213 | 0.106992 | 1.196823 | 0.338228 | 2.256338 | 0 | 0.059459 | 0 |
| RSG33856.t1 | *RsNAC117* | 0.744689 | 0.259943 | 0.496045 | 0.068046 | 0.814825 | 3.183984 | 2.792762 | 0.22466 | 0.215908 | 0.554852 | 0.115336 | 0.335583 |
| RSG36727.t1 | *RsNAC128* | 0.278443 | 0.091195 | 0.11956 | 0.116393 | 1.461011 | 8.042085 | 82.42286 | 1.215404 | 0.607149 | 0.707654 | 0.184298 | 0.668713 |
| RSG16872.t1 | *RsNAC062* | 36.12382 | 37.67906 | 19.31635 | 4.892812 | 27.74463 | 15.82394 | 13.72655 | 80.37762 | 124.0875 | 13.75788 | 53.95092 | 63.12146 |
| RSG30206.t1 | *RsNAC107* | 11.82877 | 3.284944 | 2.520171 | 1.483594 | 4.56 | 2.62163 | 9.430665 | 5.91206 | 10.77697 | 2.328175 | 5.134314 | 4.991772 |
| RSG00723.t1 | *RsNAC003* | 44.41846 | 25.72959 | 4.463346 | 3.250804 | 64.06406 | 23.71242 | 32.26901 | 58.81507 | 47.43529 | 12.85574 | 58.71719 | 40.33986 |
| RSG08857.t1 | *RsNAC027* | 232.5052 | 91.47008 | 9.084432 | 18.21898 | 125.3247 | 175.2621 | 110.4895 | 84.63511 | 62.02122 | 40.67342 | 102.9272 | 101.856 |
| RSG20033.t1 | *RsNAC077* | 68.711 | 24.00493 | 6.934603 | 3.586045 | 54.42468 | 29.01898 | 11.5002 | 22.62594 | 16.66319 | 8.419939 | 38.71726 | 21.55493 |
| RSG23615.t1 | *RsNAC088* | 66.94623 | 34.492 | 7.01822 | 3.612461 | 25.89801 | 11.43859 | 20.55565 | 37.66136 | 32.34572 | 6.654038 | 44.75941 | 35.05558 |
| RSG25363.t1 | *RsNAC092* | 73.55441 | 14.07364 | 3.150714 | 3.249174 | 10.3496 | 5.706276 | 23.18015 | 10.58208 | 15.13486 | 2.016136 | 9.327681 | 9.092995 |
| RSG30854.t1 | *RsNAC108* | 7.139168 | 13.90048 | 2.518493 | 0.958804 | 2.745365 | 7.375186 | 70.17078 | 22.08939 | 24.58303 | 1.878004 | 8.612995 | 5.232172 |
| RSG52993.t1 | *RsNAC168* | 4.580914 | 3.793802 | 1.468724 | 0.565235 | 1.325536 | 1.690073 | 17.67821 | 12.09996 | 24.88965 | 1.206507 | 5.209446 | 9.439845 |
| RSG38363.t1 | *RsNAC133* | 0.311009 | 0.110029 | 0.114014 | 0.668414 | 8.736187 | 17.20287 | 22.17909 | 13.2428 | 9.205628 | 1.549638 | 10.49157 | 10.89764 |
| RSG12244.t1 | *RsNAC048* | 35.88368 | 5.470149 | 1.148477 | 0.942709 | 12.71777 | 11.67863 | 16.10311 | 1.227175 | 1.62725 | 0.333893 | 1.962752 | 1.355102 |
|  |  |  |  |  |  |  |  |  |  |  |  |  |  |
| **Table S5**. Continued | | | | | | | | | | | | | |
| RSG39697.t1 | *RsNAC140* | 0.200276 | 0 | 0 | 0 | 0 | 0 | 0.357524 | 0 | 0.133177 | 0 | 0 | 0 |
| RSG11497.t1 | *RsNAC039* | 16.33309 | 20.4243 | 22.10221 | 17.34967 | 21.67011 | 23.95876 | 9.700835 | 9.941848 | 10.03346 | 13.61998 | 12.91363 | 13.29761 |
| RSG17948.t1 | *RsNAC066* | 1.45303 | 0.641443 | 0.470693 | 2.835781 | 2.73616 | 1.097273 | 2.686034 | 1.734703 | 0.698287 | 2.245067 | 1.600888 | 0.857815 |
| RSG11915.t1 | *RsNAC041* | 22.722 | 14.22672 | 13.74865 | 7.507784 | 13.45974 | 11.15251 | 10.00713 | 7.48705 | 9.63987 | 2.454527 | 6.383871 | 4.592838 |
| RSG22217.t1 | *RsNAC081* | 1.363496 | 0.245284 | 0.334798 | 3.699525 | 3.656941 | 6.716899 | 3.046433 | 3.599544 | 3.350795 | 2.045045 | 1.802695 | 2.537336 |
| RSG09412.t1 | *RsNAC030* | 10.45852 | 3.545039 | 2.45566 | 9.752165 | 29.26132 | 76.58014 | 4.453251 | 3.73626 | 1.455813 | 3.181076 | 3.444704 | 2.496666 |
| RSG22125.t1 | *RsNAC080* | 44.46245 | 60.30124 | 61.905 | 27.60462 | 50.25606 | 45.1044 | 22.58441 | 29.51867 | 40.2092 | 24.46979 | 43.84123 | 50.36807 |
| RSG12277.t1 | *RsNAC049* | 9.497493 | 0.621033 | 0.226407 | 0 | 0 | 0 | 0 | 0 | 0.033611 | 0 | 0 | 0 |
| RSG16488.t1 | *RsNAC061* | 12.02616 | 8.752369 | 8.549217 | 4.447357 | 6.845506 | 4.045022 | 1.71635 | 0.105475 | 0.258817 | 0.074523 | 0.232423 | 0.16297 |
| RSG13541.t1 | *RsNAC053* | 0 | 0 | 0.076009 | 0 | 0 | 0 | 0 | 0 | 0.033611 | 0 | 0 | 0 |
| RSG05699.t1 | *RsNAC022* | 0 | 0 | 0 | 0 | 0 | 0 | 0 | 0 | 0 | 0 | 0 | 0 |
| RSG45507.t1 | *RsNAC151* | 0.099531 | 0.031468 | 0 | 0 | 0.02104 | 0 | 0 | 0 | 0 | 0 | 0 | 0.081951 |
| RSG31961.t1 | *RsNAC113* | 11.16769 | 5.556488 | 4.006839 | 0.619213 | 0.274769 | 0.516067 | 0.314728 | 0 | 0 | 0 | 0 | 0 |
| RSG13739.t1 | *RsNAC054* | 14.32339 | 6.336138 | 11.16003 | 1.731197 | 1.372977 | 0.66676 | 1.382662 | 0.712332 | 2.409804 | 0.729111 | 1.419333 | 1.845052 |
| RSG51320.t1 | *RsNAC165* | 28.35613 | 6.100666 | 5.825309 | 0.875311 | 0.766998 | 0.501036 | 0.18953 | 0 | 0.034264 | 0.045518 | 0 | 0 |
| RSG17079.t1 | *RsNAC063* | 6.077277 | 2.535025 | 7.153104 | 1.617807 | 2.624683 | 5.724904 | 0.226348 | 0 | 0.036077 | 0.065879 | 0 | 0 |
| RSG32951.t1 | *RsNAC115* | 4.794261 | 2.100296 | 8.226379 | 1.842817 | 1.878175 | 0.663162 | 0.03571 | 0 | 0 | 0 | 0 | 0 |
| RSG09835.t1 | *RsNAC031* | 0.254442 | 0.037899 | 0.20154 | 0 | 0 | 0 | 0 | 0.367131 | 0.578565 | 0.625354 | 2.84801 | 3.791964 |
| RSG51048.t1 | *RsNAC162* | 0.225017 | 0.615158 | 0.55006 | 4.06673 | 0.376915 | 0.620952 | 0.035376 | 0.836196 | 0.262107 | 0.907868 | 0.630861 | 0.555523 |
| RSG19372.t1 | *RsNAC072* | 5.625907 | 3.083852 | 1.732579 | 0.250048 | 0.577792 | 0.364652 | 10.36998 | 2.624698 | 1.410907 | 0.421368 | 1.240881 | 0.942037 |
| RSG51109.t1 | *RsNAC163* | 0.562644 | 0.074183 | 0.25627 | 0.03808 | 0 | 0 | 0.144371 | 0.181099 | 0.032378 | 0 | 0 | 0 |
| RSG21630.t1 | *RsNAC079* | 51.40072 | 38.19706 | 21.36353 | 10.77159 | 6.399254 | 2.838345 | 1.418715 | 2.68491 | 0.746379 | 0.951169 | 0.545919 | 0.329368 |
| RSG26111.t1 | *RsNAC095* | 3.247264 | 1.294757 | 0.707488 | 0.567602 | 0.386609 | 0.261407 | 0.218038 | 0.429147 | 0.203072 | 0.440537 | 0 | 0.134044 |
| RSG23023.t1 | *RsNAC086* | 9.643231 | 8.18799 | 4.664164 | 3.329909 | 6.527709 | 5.414036 | 0.383803 | 0.080265 | 0 | 0.216007 | 0.145683 | 0.323045 |
| RSG28433.t1 | *RsNAC103* | 8.790367 | 6.77083 | 1.24885 | 0.431045 | 3.074571 | 5.912379 | 17.05275 | 0.139743 | 0.239594 | 0.320629 | 0.157463 | 0.043088 |
| RSG29844.t1 | *RsNAC106* | 0.891763 | 0.272778 | 0.051295 | 0.07924 | 0.058328 | 0.09595 | 14.90996 | 0.450682 | 0.939916 | 0.539846 | 0.32105 | 0.411214 |
| RSG08804.t1 | *RsNAC026* | 4.00668 | 3.19368 | 0.809529 | 1.346274 | 2.550362 | 4.093569 | 1.506538 | 1.322883 | 0.94509 | 0.797213 | 0.894347 | 0.668288 |
| RSG23385.t1 | *RsNAC087* | 7.204212 | 1.65252 | 0.434315 | 1.258604 | 0.80574 | 3.191153 | 2.041105 | 0.46318 | 0.398133 | 0.127339 | 0.047313 | 0.129549 |
| RSG52719.t1 | *RsNAC166* | 8.12535 | 10.60318 | 14.72569 | 16.43365 | 66.16228 | 34.31513 | 11.47494 | 4.535033 | 4.782053 | 8.949752 | 5.275064 | 5.332971 |
| RSG20769.t1 | *RsNAC078* | 0.78808 | 5.479912 | 2.658512 | 6.117038 | 8.495004 | 10.27379 | 1.068451 | 1.413149 | 0.317863 | 0.928513 | 0.886443 | 0.709294 |
|  |  |  |  |  |  |  |  |  |  |  |  |  |  |
| **Table S5**. Continued | | | | | | | | | | | | | |
| RSG27552.t1 | *RsNAC096* | 0.028624 | 0.130271 | 0.053804 | 0.319713 | 0.046309 | 0.113244 | 0.051037 | 0.053231 | 0.023792 | 0 | 0.09246 | 0.063825 |
| RSG11079.t1 | *RsNAC037* | 30.38743 | 20.86891 | 6.475397 | 4.274023 | 1.995103 | 4.515337 | 0.998802 | 0 | 0.097697 | 0.05096 | 0 | 0.191691 |
| RSG01200.t1 | *RsNAC004* | 0.139829 | 0.170383 | 0.318284 | 4.667496 | 2.631037 | 2.648088 | 0 | 0 | 0 | 0.078139 | 0 | 0 |
| RSG08749.t1 | *RsNAC025* | 0.649749 | 0 | 0.043211 | 1.091764 | 0.203328 | 0.407771 | 0 | 0 | 0 | 0.063182 | 0.028114 | 0.066589 |
| RSG17257.t1 | *RsNAC064* | 0.191608 | 0.227485 | 0.1398 | 0.681049 | 0.189726 | 0.394169 | 0.220239 | 0.171031 | 0.167592 | 0 | 0.015868 | 0.087124 |
| RSG46630.t1 | *RsNAC154* | 0.213119 | 0.072628 | 0 | 0 | 0 | 0 | 0 | 0.035461 | 0 | 0 | 0 | 0 |
| RSG34595.t1 | *RsNAC119* | 1.207527 | 1.38655 | 0.734291 | 9.532942 | 3.18863 | 2.260969 | 0 | 0 | 0 | 0.038961 | 0 | 0 |
| RSG39304.t1 | *RsNAC138* | 0.372078 | 1.529969 | 3.296171 | 16.80097 | 2.715163 | 5.686042 | 0.318206 | 1.065985 | 0.590538 | 1.00585 | 0.700497 | 0.30417 |
| RSG14554.t1 | *RsNAC056* | 0 | 0 | 0.046496 | 0 | 0 | 0 | 0 | 0.311986 | 0.22663 | 0 | 0.106854 | 0 |
| RSG17700.t1 | *RsNAC065* | 0.132926 | 0.032279 | 0.148756 | 0 | 0 | 0 | 0 | 0 | 0 | 0 | 0 | 0 |
| RSG33885.t1 | *RsNAC118* | 1.582251 | 0.368345 | 0 | 0 | 0 | 0 | 0.065331 | 0 | 0 | 0 | 0 | 0.033846 |
| RSG14848.t1 | *RsNAC057* | 2.984603 | 6.450276 | 3.754812 | 8.10813 | 1.637844 | 1.091165 | 2.059809 | 2.419023 | 0.614984 | 1.238229 | 1.580948 | 1.467943 |
| RSG28764.t1 | *RsNAC105* | 1.29925 | 3.05315 | 2.530132 | 4.803298 | 0.65512 | 0.652059 | 0.52908 | 0.72911 | 0.235171 | 0.976107 | 0.801506 | 0.521537 |
| RSG15654.t1 | *RsNAC059* | 0.827334 | 1.081597 | 1.247881 | 2.547112 | 1.172654 | 4.702667 | 0.577401 | 0.559125 | 0.094115 | 1.412951 | 0.863798 | 0.927488 |
| RSG28351.t1 | *RsNAC099* | 0.488037 | 1.573929 | 1.093037 | 2.189251 | 0.598177 | 1.110497 | 0.293244 | 0.788319 | 0.30013 | 0.393518 | 0.331363 | 0.633228 |
| RSG12456.t1 | *RsNAC050* | 1.236339 | 3.193856 | 4.554302 | 2.56411 | 1.300943 | 0.832023 | 0.83279 | 2.800552 | 0.301779 | 2.959928 | 1.710628 | 1.391159 |
| RSG49510.t1 | *RsNAC159* | 0.838826 | 1.544026 | 1.906189 | 1.958737 | 0.63945 | 0.591811 | 0.501201 | 1.387459 | 0.297641 | 0.77752 | 0.641056 | 0.496938 |
| RSG46518.t1 | *RsNAC153* | 0.426317 | 0.723889 | 0.749141 | 2.153448 | 0.754228 | 0.631865 | 0.03571 | 0.613193 | 0.071926 | 0.689638 | 0.610729 | 0.249211 |
| RSG04122.t1 | *RsNAC020* | 1.262404 | 1.485066 | 0.702618 | 1.102083 | 1.782634 | 1.648652 | 1.98341 | 5.078058 | 4.38383 | 3.268021 | 3.447858 | 3.228451 |
| RSG08956.t1 | *RsNAC029* | 0.49568 | 2.352887 | 1.699111 | 2.718971 | 1.956449 | 1.220774 | 1.57428 | 3.083075 | 2.084211 | 5.476381 | 6.187787 | 3.545182 |
| RSG10550.t1 | *RsNAC035* | 2.168888 | 6.202923 | 5.131873 | 9.787017 | 5.171459 | 22.01867 | 2.62847 | 2.771912 | 2.300287 | 4.488065 | 4.600151 | 5.456759 |
| RSG45857.t1 | *RsNAC152* | 0.373775 | 0.681693 | 0.980494 | 1.384396 | 0.616645 | 0.095318 | 0.258607 | 2.586652 | 1.382068 | 2.632012 | 2.469285 | 2.012572 |
| RSG01548.t1 | *RsNAC006* | 14.9226 | 6.68124 | 10.38525 | 3.09385 | 2.868398 | 1.857587 | 1.696769 | 1.409759 | 0.387081 | 0.7877 | 0.740223 | 1.366392 |
| RSG01805.t1 | *RsNAC008* | 19.90592 | 11.52175 | 9.008383 | 13.06217 | 11.79479 | 10.7912 | 3.457963 | 5.419063 | 8.062611 | 2.259324 | 1.252783 | 2.122975 |
| RSG13466.t1 | *RsNAC052* | 87.02453 | 83.53957 | 37.15939 | 31.27758 | 99.99563 | 68.48171 | 66.89986 | 88.20198 | 70.51469 | 83.44028 | 189.5192 | 115.2985 |
| RSG18153.t1 | *RsNAC067* | 6.611673 | 5.590353 | 0.634922 | 0.186015 | 7.30952 | 0.682945 | 6.124694 | 14.56351 | 21.54141 | 89.84158 | 102.4053 | 39.73063 |
| RSG35036.t1 | *RsNAC123* | 0.981415 | 0.444966 | 0.536245 | 1.397295 | 11.40257 | 3.898342 | 7.362042 | 7.957873 | 19.77667 | 3.480174 | 9.831423 | 6.460831 |
| RSG19414.t1 | *RsNAC073* | 2.60717 | 0.146861 | 0.067379 | 0.025662 | 3.682694 | 0.134648 | 4.060375 | 1.175995 | 0.954711 | 14.19259 | 19.68522 | 3.25792 |
| RSG02101.t1 | *RsNAC010* | 0.647596 | 0.131957 | 0.042603 | 0.066936 | 0.330066 | 0.039602 | 1.270268 | 1.176051 | 0.955047 | 0.670889 | 6.254727 | 4.2139 |
| RSG11762.t1 | *RsNAC040* | 26.17394 | 26.82101 | 25.02582 | 23.7137 | 30.66851 | 31.64091 | 15.6811 | 13.9637 | 10.9731 | 14.93505 | 18.51247 | 19.02157 |
|  |  |  |  |  |  |  |  |  |  |  |  |  |  |
| **Table S5**. Continued | | | | | | | | | | | | | |
| RSG10501.t1 | *RsNAC034* | 3.029425 | 3.351073 | 3.256292 | 5.758974 | 5.757438 | 6.496244 | 1.699884 | 2.029983 | 1.935686 | 1.274686 | 1.55627 | 1.186537 |
| RSG07643.t1 | *RsNAC023* | 0.035308 | 0 | 0 | 0.072097 | 0.530766 | 2.322361 | 0 | 0 | 0 | 0 | 0 | 0.078399 |
| RSG19420.t1 | *RsNAC074* | 0.489077 | 3.687499 | 3.275691 | 0.298724 | 0.442042 | 0.107818 | 0.31206 | 0.156533 | 0.033978 | 0 | 0 | 0 |
| RSG18158.t1 | *RsNAC068* | 0.466292 | 0.037899 | 0 | 0 | 0 | 0 | 0.036455 | 0 | 0 | 0 | 0 | 0 |
| RSG35030.t1 | *RsNAC122* | 0.034091 | 0 | 0 | 0.053185 | 0.144868 | 0 | 0 | 0.03539 | 0 | 0.06001 | 0 | 0 |
| RSG14996.t1 | *RsNAC058* | 157.0092 | 66.17641 | 31.7471 | 20.14273 | 91.34104 | 44.79675 | 104.1852 | 29.03677 | 24.23605 | 45.13378 | 105.7201 | 88.47787 |
| RSG16412.t1 | *RsNAC060* | 61.92589 | 106.5065 | 47.65597 | 36.07445 | 88.86905 | 54.11272 | 28.10268 | 76.81012 | 57.20864 | 122.4847 | 257.4929 | 132.9683 |
| RSG53660.t1 | *RsNAC169* | 2.183363 | 0.081751 | 0.337775 | 0.044001 | 0.100253 | 0.039322 | 0.207103 | 0.122586 | 0 | 0 | 0.036558 | 0.186163 |
| RSG10766.t1 | *RsNAC036* | 10.30375 | 12.72469 | 15.26922 | 13.36018 | 8.799279 | 5.81091 | 4.24716 | 8.506783 | 3.97934 | 3.217858 | 2.60393 | 3.890199 |
| RSG44690.t1 | *RsNAC149* | 3.139299 | 2.96371 | 3.666912 | 5.04357 | 5.31719 | 3.627853 | 2.026165 | 3.4121 | 1.56707 | 2.058319 | 1.465363 | 1.89755 |
| RSG36770.t1 | *RsNAC129* | 3.609975 | 4.722861 | 2.252372 | 3.418849 | 2.487069 | 2.787369 | 0.984985 | 2.030957 | 0.539648 | 1.400048 | 0.952574 | 1.284669 |
| RSG02614.t1 | *RsNAC013* | 0.124923 | 0.20981 | 0.061679 | 0.583683 | 2.09772 | 1.132013 | 0.079292 | 0 | 0 | 0.049007 | 0.046639 | 0 |
| RSG18827.t1 | *RsNAC069* | 5.105875 | 23.26049 | 29.48641 | 54.5351 | 37.70389 | 20.0903 | 0 | 0.119627 | 0.053545 | 0.072012 | 0.141878 | 0 |
| RSG28487.t1 | *RsNAC104* | 4.195241 | 3.374332 | 2.679486 | 1.328536 | 3.472874 | 5.165309 | 0.191172 | 0.898649 | 0.074154 | 0.078286 | 0.017417 | 0 |
| RSG01327.t1 | *RsNAC005* | 0.816619 | 1.536147 | 1.290503 | 3.135053 | 1.105323 | 0.57956 | 0.535057 | 1.180941 | 0.384187 | 0.322568 | 0.135434 | 0.498759 |
| RSG53820.t1 | *RsNAC170* | 0.492297 | 0.856277 | 0.284249 | 1.250676 | 0.552874 | 0.775347 | 0.306848 | 0.687399 | 0.704036 | 0 | 0.03359 | 0 |
| RSG12094.t1 | *RsNAC046* | 3.805894 | 2.685432 | 2.242788 | 5.963505 | 1.802957 | 2.748754 | 1.977191 | 3.848605 | 1.933711 | 0.437168 | 0.434764 | 0.402162 |
| RSG31855.t1 | *RsNAC112* | 0.914212 | 1.565161 | 0.461975 | 0.81187 | 1.012873 | 0.527972 | 1.113201 | 2.284137 | 1.057005 | 0.44732 | 0.233279 | 0.425251 |
| RSG01763.t1 | *RsNAC007* | 62.01107 | 81.7336 | 64.56339 | 61.77777 | 29.54558 | 32.19841 | 73.70481 | 65.67058 | 92.90914 | 12.3865 | 16.90162 | 17.14769 |
| RSG14482.t1 | *RsNAC055* | 18.73611 | 29.46274 | 33.77106 | 41.25851 | 38.67262 | 43.27375 | 91.1274 | 27.76719 | 47.17075 | 20.81138 | 17.02073 | 32.61661 |
| RSG05613.t1 | *RsNAC021* | 67.0007 | 70.21381 | 30.06738 | 51.53003 | 27.41471 | 26.09567 | 131.909 | 58.13225 | 48.71973 | 22.35113 | 17.79272 | 29.33682 |
| RSG48570.t1 | *RsNAC157* | 15.09992 | 13.89156 | 13.15664 | 11.59309 | 14.47261 | 14.85175 | 11.37757 | 7.441606 | 6.727417 | 8.366172 | 8.681738 | 8.677949 |
| RSG48659.t1 | *RsNAC158* | 18.98454 | 6.926495 | 2.78289 | 1.822148 | 6.118435 | 3.167477 | 0.849127 | 0.220299 | 0.233155 | 0.263672 | 0.154783 | 0.251194 |
| RSG52916.t1 | *RsNAC167* | 15.09042 | 12.69799 | 13.6236 | 12.46117 | 15.57793 | 15.95018 | 16.44541 | 9.798042 | 8.026804 | 12.22839 | 8.30249 | 8.809257 |
| RSG35848.t1 | *RsNAC126* | 23.06869 | 13.00011 | 9.560083 | 7.018569 | 15.74375 | 14.98194 | 11.96814 | 2.933911 | 3.117014 | 3.819127 | 4.059056 | 3.537074 |
| RSG19838.t1 | *RsNAC075* | 22.96585 | 12.3648 | 9.472239 | 10.24081 | 84.8568 | 67.47057 | 26.21737 | 51.07185 | 57.153 | 70.35044 | 183.7043 | 101.9519 |
| RSG38993.t1 | *RsNAC137* | 0.258055 | 0.0406 | 0.166799 | 0.173882 | 0.157959 | 0.828121 | 9.56303 | 4.440499 | 20.64614 | 0.40699 | 5.392635 | 8.149527 |
| RSG03030.t1 | *RsNAC016* | 12.68164 | 11.12978 | 2.97292 | 3.405875 | 20.99893 | 15.32435 | 31.02393 | 28.80096 | 28.36645 | 22.01039 | 78.76534 | 47.7247 |
| RSG22781.t1 | *RsNAC084* | 3.028954 | 4.775786 | 1.268435 | 0.015216 | 0.071286 | 0.046775 | 5.457968 | 11.12099 | 15.7053 | 7.914418 | 7.337695 | 8.432278 |
| RSG27764.t1 | *RsNAC097* | 6.762756 | 4.028601 | 0.692309 | 0 | 0.018619 | 0 | 9.723652 | 21.66959 | 12.80979 | 24.64154 | 12.78376 | 16.14329 |
|  |  |  |  |  |  |  |  |  |  |  |  |  |  |
| **Table S5**. Continued | | | | | | | | | | | | | |
| RSG03678.t1 | *RsNAC017* | 0.510247 | 0.592859 | 0.201562 | 0.811567 | 5.279082 | 17.76667 | 0.03738 | 0.149823 | 0.262441 | 0 | 0.087773 | 0.046196 |
| RSG11087.t1 | *RsNAC038* | 0 | 0.26715 | 0.03837 | 0.019411 | 0.082838 | 0.119337 | 0.078519 | 0.073231 | 0.170129 | 0 | 0 | 0.03927 |
| RSG18911.t1 | *RsNAC070* | 1.840961 | 0.740944 | 0.541934 | 0 | 0.141659 | 0 | 2.844697 | 0.258175 | 0.038925 | 0 | 0.019018 | 0.045045 |
| RSG35960.t1 | *RsNAC127* | 0.537237 | 0.584675 | 0.806433 | 0.024618 | 0.162464 | 0 | 0.33076 | 0.048146 | 0 | 0 | 0.021028 | 0.057276 |
| RSG55051.t1 | *RsNAC171* | 19.19161 | 11.09736 | 4.133839 | 0 | 0.452043 | 0.417205 | 2.859554 | 0.915059 | 1.558384 | 0.636539 | 0.963053 | 1.408554 |
| RSG24155.t1 | *RsNAC089* | 8.821527 | 2.73886 | 1.45365 | 0.043569 | 0.138602 | 0 | 3.387735 | 1.996279 | 4.358071 | 0.537439 | 1.580661 | 0.800108 |
| RSG31027.t1 | *RsNAC109* | 0.105925 | 0 | 0 | 0 | 0 | 0 | 0 | 0 | 0 | 0 | 0 | 0 |
| RSG32015.t1 | *RsNAC114* | 0.031302 | 0 | 0.031587 | 0 | 0 | 0 | 0 | 0 | 0.040971 | 0 | 0 | 0 |
| RSG27884.t1 | *RsNAC098* | 0 | 0.075564 | 0.10348 | 0 | 0 | 0 | 0 | 0 | 0 | 0 | 0 | 0 |
| RSG38941.t1 | *RsNAC135* | 0 | 0 | 0.02864 | 0 | 0 | 0 | 0 | 0 | 0 | 0 | 0 | 0 |
| RSG10059.t1 | *RsNAC032* | 41.45261 | 38.00509 | 5.436218 | 1.155324 | 1.268894 | 0.976057 | 8.947999 | 10.09106 | 10.52467 | 12.63966 | 4.807143 | 6.609868 |
| RSG22714.t1 | *RsNAC083* | 20.09067 | 3.525647 | 0.966337 | 0.02375 | 0.060894 | 0 | 0.322589 | 0.170353 | 0.431803 | 0.54989 | 0.111105 | 0.144145 |
| RSG02694.t1 | *RsNAC014* | 22.82003 | 21.46258 | 29.76876 | 30.44633 | 36.0304 | 39.28841 | 14.41519 | 14.30674 | 13.2139 | 23.77872 | 17.30448 | 20.63288 |
| RSG25336.t1 | *RsNAC091* | 3.400253 | 5.270352 | 5.028325 | 5.925365 | 7.30748 | 2.264512 | 1.608036 | 4.413014 | 2.896944 | 7.347342 | 6.119569 | 7.290043 |
| RSG08863.t1 | *RsNAC028* | 38.10868 | 25.8391 | 29.46204 | 26.93652 | 39.53309 | 31.04447 | 13.33147 | 11.83292 | 12.84035 | 9.812492 | 8.942146 | 7.827221 |
| RSG20023.t1 | *RsNAC076* | 54.15495 | 43.91536 | 34.56985 | 25.67246 | 57.55342 | 54.36772 | 29.98262 | 36.03308 | 30.83646 | 35.58556 | 70.33729 | 52.59353 |
| RSG49516.t1 | *RsNAC161* | 0 | 0 | 0 | 0 | 0 | 0 | 0 | 0 | 0 | 0.200928 | 0 | 0.067094 |
| RSG31666.t1 | *RsNAC111* | 12.94122 | 12.44852 | 15.36672 | 14.06145 | 16.50234 | 23.14002 | 6.256622 | 4.729159 | 3.999169 | 10.90197 | 5.962731 | 8.118167 |
| RSG48380.t1 | *RsNAC156* | 20.84154 | 40.12913 | 56.8857 | 41.14322 | 109.4848 | 160.4333 | 9.685394 | 11.15771 | 9.521753 | 4.001518 | 5.227193 | 13.5689 |
| RSG43885.t1 | *RsNAC144* | 0 | 0 | 0 | 0 | 0.230626 | 0 | 0 | 0 | 0 | 0 | 0 | 0 |
| RSG28379.t1 | *RsNAC100* | 40.57186 | 20.9217 | 16.07934 | 12.028 | 49.01845 | 41.35906 | 38.03669 | 10.74862 | 5.790958 | 8.691073 | 36.35736 | 31.11994 |
| RSG35391.t1 | *RsNAC124* | 17.89128 | 7.360253 | 5.905942 | 6.326155 | 9.124817 | 10.76 | 4.16198 | 4.093076 | 5.670412 | 2.521249 | 2.131006 | 5.135693 |
| RSG25670.t1 | *RsNAC093* | 0.168501 | 0 | 0 | 0 | 0 | 0 | 0 | 0 | 0.053179 | 0 | 0 | 0 |
| RSG25672.t1 | *RsNAC094* | 0.041193 | 0.040013 | 0 | 0 | 0.116151 | 0.651072 | 0 | 0 | 0 | 0 | 0 | 0 |
| RSG28382.t1 | *RsNAC101* | 1.491147 | 0.827139 | 1.67286 | 2.406664 | 2.368869 | 2.931822 | 0.1375 | 0 | 0 | 0 | 0.030075 | 0 |
| RSG28385.t1 | *RsNAC102* | 13.31554 | 18.76149 | 15.39883 | 2.575111 | 17.26996 | 11.1822 | 11.32145 | 8.657075 | 5.858408 | 3.759685 | 42.58586 | 16.27385 |
