# Supplementary material for: Genome-wide characterization and expression profiling of NAC transcription factor genes under abiotic stresses in radish (Raphanus sativus L.)
Source: PeerJ. 2017 Dec 15;5:e4172. doi: 10.7717/peerj.4172 (PMC5733918; doi:10.7717/peerj.4172)
Supplement: Table S6 — Fold change values obtained from transcript levels of 21 randomly selected RsNAC genes using real-time polymerase chain reaction. aPolyethylene glycol. babscisic acid. [file peerj-05-4172-s010.docx]

| **Table S6.** The fold change values of *RsNAC* genes under abiotic stresses | | | | |  |  |
| --- | --- | --- | --- | --- | --- | --- |
| **Gene name** | **Treatment** | | | |  |  |
|  | **Cadmium(Cd)** | **Lead (Pb)** | **Salt** | **Heat** | **^a^PEG** | **^b^ABA** |
| *RsNAC008* | 2.00 | 5.00 | 4.00 | 0.50 | 1.25 | 0.31 |
| *RsNAC023* | 0.08 | 0.06 | 0.25 | 0.08 | 0.16 | 0.16 |
| *RsNAC027* | 4.00 | 5.98 | 3.99 | 1.57 | 0.18 | 0.19 |
| *RsNAC034* | 0.15 | 0.15 | 0.24 | 0.49 | 0.49 | 0.50 |
| *RsNAC038* | 12.65 | 10.14 | 15.87 | 1.40 | 1.50 | 1.44 |
| *RsNAC039* | 0.79 | 0.64 | 1.60 | 2.52 | 2.01 | 0.79 |
| *RsNAC040* | 0.12 | 0.15 | 0.11 | 0.50 | 0.79 | 1.63 |
| *RsNAC041* | 0.08 | 0.05 | 0.50 | 0.63 | 0.63 | 0.16 |
| *RsNAC055* | 3.90 | 3.70 | 2.02 | 0.20 | 1.56 | 0.12 |
| *RsNAC058* | 1.23 | 0.39 | 0.98 | 3.08 | 1.23 | 0.19 |
| *RsNAC062* | 2.00 | 4.00 | 5.00 | 4.00 | 2.00 | 2.00 |
| *RsNAC080* | 2.47 | 1.56 | 1.98 | 7.82 | 1.95 | 0.50 |
| *RsNAC096* | 0.30 | 0.32 | 0.24 | 0.15 | 0.16 | 0.22 |
| *RsNAC103* | 0.51 | 0.04 | 0.40 | 0.04 | 0.20 | 0.00 |
| *RsNAC124* | 0.08 | 0.02 | 0.29 | 0.29 | 0.29 | 0.05 |
| *RsNAC126* | 4.02 | 4.00 | 1.00 | 0.40 | 0.22 | 0.24 |
| *RsNAC140* | 1.00 | 0.20 | 0.25 | 1.57 | 0.20 | 0.32 |
| *RsNAC145* | 0.39 | 0.31 | 0.78 | 1.97 | 1.58 | 2.02 |
| *RsNAC156* | 0.08 | 0.02 | 0.29 | 0.29 | 0.29 | 0.05 |
| *RsNAC158* | 0.03 | 0.03 | 0.07 | 0.06 | 0.26 | 0.07 |
| *RsNAC168* | 0.20 | 0.16 | 5.08 | 1.59 | 1.00 | 0.08 |
